# Histone acetyltransferase KAT2A modulates neural stem cell differentiation and proliferation by inducing degradation of the transcription factor PAX6

**DOI:** 10.1016/j.jbc.2023.103020

**Published:** 2023-02-13

**Authors:** Zhangji Dong, Wei He, Ge Lin, Xu Chen, Sixian Cao, Tuchen Guan, Ying Sun, Yufang Zhang, Mengwei Qi, Beibei Guo, Zhihao Zhou, Run Zhuo, Ronghua Wu, Mei Liu, Yan Liu

**Affiliations:** Key Laboratory of Neuroregeneration of Jiangsu and Ministry of Education, Co-innovation Center of Neuroregeneration, NMPA Key Laboratory for Research and Evaluation of Tissue Engineering Technology Products, Nantong University, Nantong, Jiangsu, China

**Keywords:** KAT2A, PAX6, neural stem cells, differentiation, ubiquitination, acetylation, CBP, CREB-binding protein, DMSO, dimethyl sulfoxide, HAT, histone acetyltransferase, MO, morpholino oligonucleotide, MS/MS, tandem mass spectrometry, NSC, neural stem cell, PAX6, paired box 6, RNF8, ring finger protein 8, SOX2, SRY-box transcription factor 2, TRIM11, tripartite motif containing 11

## Abstract

Neural stem cells (NSCs) proliferation and differentiation rely on proper expression and posttranslational modification of transcription factors involved in the determination of cell fate. Further characterization is needed to connect modifying enzymes with their transcription factor substrates in the regulation of these processes. Here, we demonstrated that the inhibition of KAT2A, a histone acetyltransferase, leads to a phenotype of small eyes in the developing embryo of zebrafish, which is associated with enhanced proliferation and apoptosis of NSCs in zebrafish eyes. We confirmed that this phenotype is mediated by the elevated level of PAX6 protein. We further verified that KAT2A negatively regulates PAX6 at the protein level in cultured neural stem cells of rat cerebral cortex. We revealed that PAX6 is a novel acetylation substrate of KAT2A and the acetylation of PAX6 promotes its ubiquitination mediated by the E3 ligase RNF8 that facilitated PAX6 degradation. Our study proposes that KAT2A inhibition results in accelerated proliferation, delayed differentiation, or apoptosis, depending on the context of PAX6 dosage. Thus, the KAT2A/PAX6 axis plays an essential role to keep a balance between the self-renewal and differentiation of NSCs.

The developmental stages of the neural lineage cells are usually defined by the stage-specific markers including transcription factors. These transcription factors are responsible for the self-renewal, proliferation, neurogenesis, and astrogliogenesis of different neural stem cells (NSCs) or progenitors (1, 2). For example, SRY-Box transcription factor 2 (SOX2) and Paired Box 6 (PAX6) are essential for self-renewal or proliferation (3, 4), while Neurogenin 2 and Achaete-Scute Family BHLH Transcription Factor 1 (ASCL1) are important for neurogenesis (5, 6). How these factors are modulated is among the core issues in developmental neuroscience.

The levels of the transcription factors that determine the fate of NSCs are orchestrated by a complex regulatory network that extends from the epigenetic to the posttranslational level (7, 8, 9). The modification of transcription factors, including phosphorylation, acetylation, and ubiquitination, are reported to play an important role in the homeostasis of stem cells (10). Recent studies have shed light on the importance of lysine acetylation in transcription factors. Acetylation of transcription factors affects their biological role such as transcriptional activity, subcellular distribution, and stability (11). Protein acetylation on lysine residues is mediated by histone acetyltransferases (HATs), which were originally discovered as an enzyme to modify histone (11). However, the acetylation of nonhistone protein, including transcription factors, has been shown to be a ubiquitous phenomenon in eukaryotic cells. Lysine acetyltransferase 2A (KAT2A), encoded by the *Kat2a* gene, is the first histone acetyltransferase that links histone acetylation to chromatin remodeling, which results in transcriptional activation of the target gene (12). KAT2A contains a conserved HAT domain and a C-terminal bromodomain (13). Recent studies show that KAT2A is also an acetyltransferase of transcription factors, which could regulate the transcription activity or stability of the substrates (14, 15).

In this study, we found a novel substrate of KAT2A, PAX6, a transcription factor that is essential for the normal development of the brain and eyes in vertebrates (16). *Pax6*-null embryos die soon after birth, and *Pax6* heterozygous mutant mice/rats show a small eye phenotype (17). Many studies described diverse functions of *Pax6* in NSCs proliferation. In mammalian eyes, *Pax6* is required for the proliferation and expansion of retinal stem cells (18), and *Pax6* is also essential for neuronal progenitor cell proliferation during zebrafish photoreceptor regeneration (19). At early stages of cortical development (E12.5) in mice, loss of *Pax6* results in a shortening of the cell cycle of progenitors, while at the mid-corticogenesis stage (E15.5), Pax6-null progenitor cells proliferate more slowly than wildtype controls, which indicates that the effects of *Pax6* on the proliferation of cortical progenitors are context dependent. Mi *et al*. (20) further revealed that *Pax6* inhibits cortical progenitor proliferation *via* directly repressing *Cdk6* expression. Moreover, conditional activation of *Pax6* in the developing cortex is reported to cause progenitor apoptosis in mice (21). These studies proposed that *Pax6* exerts a complicated role on NSCs in a dosage- and context-dependent manner. Therefore, it is well recognized that the spatial-temporally orchestrated *Pax6* level is critical for the development of the nervous system.

In this study, we found that KAT2A inhibition gives rise to a phenotype of small eyes accompanied by an increased PAX6 level during zebrafish development. This result linked a HAT to the PAX6 level that was also verified in *in vitro* cultured NSCs. Interestingly, we found that KAT2A inhibition did not affect the mRNA level of *Pax6* in NSCs, which usually occurred in the KAT2A-mediated transcriptional regulation. However, the protein level of PAX6 in NSCs increased upon treatment with a KAT2A inhibitor. Then, we tried to explain the underlying mechanism and revealed that KAT2A could acetylate PAX6 and facilitate the ubiquitination-mediated degradation of PAX6. We further identified ring finger protein 8 (RNF8) as the E3 ligase that is responsible for the PAX6 ubiquitination. Our findings provide insight into the mechanism of the proliferation and differentiation of NSCs through posttranslational modifications on PAX6.

## Results

### Kat2a knockdown affects eye development in zebrafish

**Figure 1:**
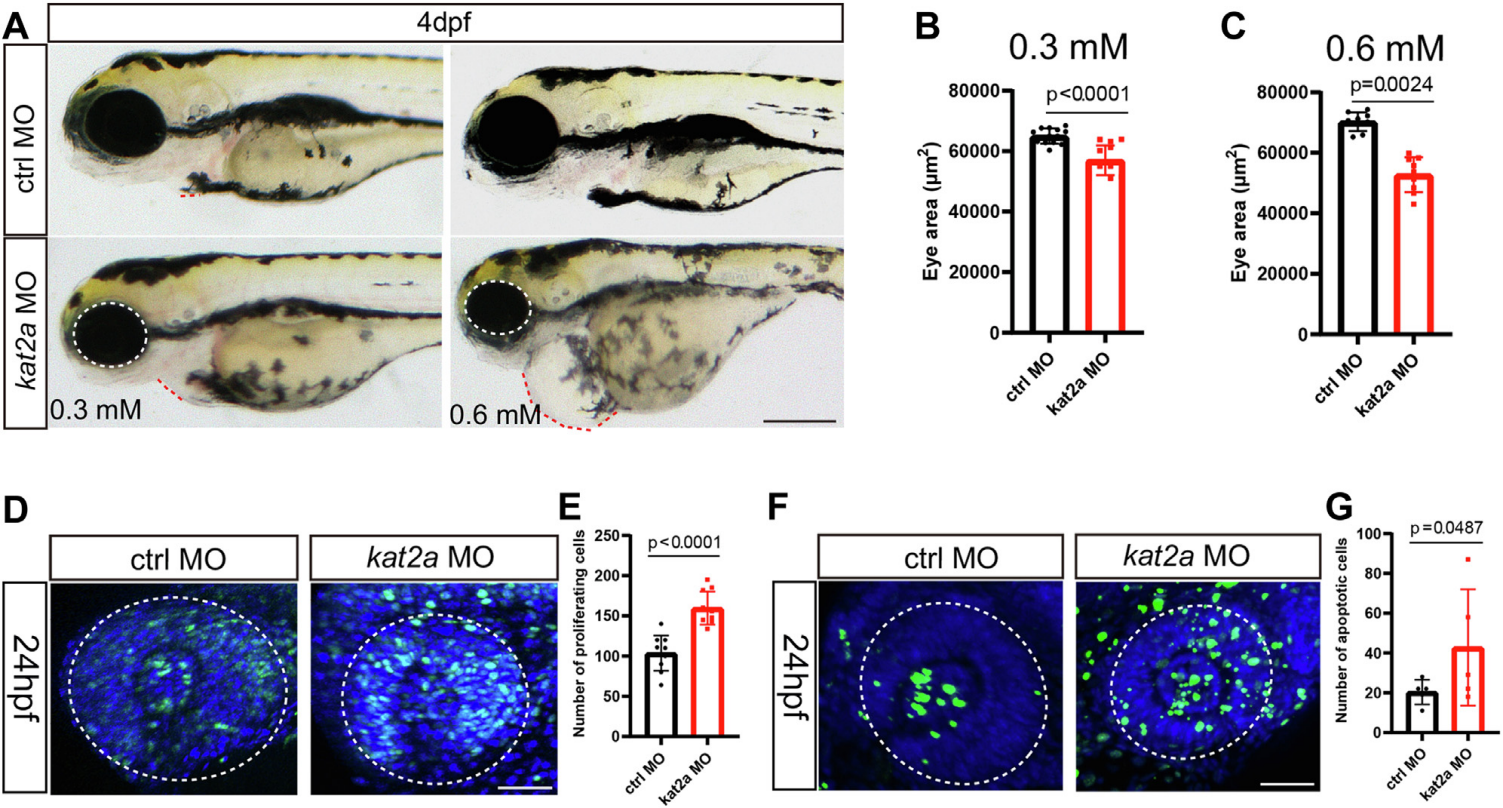
**Loss of function of *kat2a* lead to smaller eyes and increased both proliferation and apoptosis in zebrafish.***A*, the size of eyes was reduced in zebrafish morphants in a dose-dependent manner. The scale bar represents 200 μm. *B* and *C*, statistical diagrams of eye sizes of 0.3 mM *kat2a* MO-treated zebrafish and 0.6 mM *kat2a* MO-treated zebrafish, compared with siblings that received control MO injection. n = 13, 13, 9, 9 zebrafish embryos, Student’s *t* test. *D* and *E*, there were more proliferating cells in 0.3 mM *kat2a* MO-treated zebrafish at 24 hpf compared with control siblings, detected by BrdU assay. The scale bar represents 50 μm. n = 10 and 10 zebrafish embryos, Student’s *t* test. *F* and *G*, there were more apoptotic cells in 0.3 mM *kat2a* MO-treated zebrafish at 24 hpf compared with control siblings, detected by TUNEL assay. The scale bar represents 50 μm. n = 5 and 5 zebrafish embryos, Student’s *t* test. MO, morpholino oligonucleotides; TUNEL, terminal deoxynucleotidyl transferase-mediated dUTP nick-end labeling.

The roles of Kat2a during development have been defined through genetic studies in mice and other vertebrates (22, 23). Loss of Kat2a is usually embryonic lethal. Therefore, the knockdown or conditional knockout strategies might be applied for observing the function of Kat2a during neural development. Martínez-Cerdeno *et al.* (24) reported that mice with conditional null *kat2a* in Nestin-positive cells exhibit impaired brain growth and microcephaly. In this study, we investigated the effects of *kat2a* knockdown on zebrafish development using 0.3 mmol or 0.6 mmol splicing-blocking morpholino oligonucleotides (MO) (Fig. 1). Along with previously described phenotypes in the heart and limb, abnormalities were also found in the developing eyes in *kat2a* knockdown morphants. The eyes were smaller and less pigmented compared with controls (Fig. 1, *A*–*C*). The developing eyes are composed of many NSCs at 24 hpf, and the developing retinae and lens could be observed (25, 26) at this stage. The normal development of the eyes requires optimal cell proliferation. Either insufficient or excessive proliferation could give rise to an abnormal phenotype. The BrdU incorporation assay showed a significant increase in cell proliferation in the eyes of zebrafish treated with *kat2a* MO (Fig. 1, *D* and *E*), indicating the phenotype might be due to exhausted NSCs in the developing zebrafish. We further speculated whether *kat2a* knockdown gives rise to more cell death. We utilized terminal deoxynucleotidyl transferase-mediated dUTP nick-end labeling (TUNEL) to detect apoptosis in the developing eyes and observed an increase in the number of apoptotic cells in developing eyes with reduced *kat2a* expression (Fig. 1, *F* and *G*). These data showed that both proliferation and apoptosis were augmented in *kat2a* morphants, which proposed that early exhaustion of NSCs might occur in developing eyes. Then, the phenotype of small eyes might be the result of overproliferation and subsequent apoptosis of NSCs during development.

### Kat2a modulates Pax6 level during zebrafish eye development

**Figure 2:**
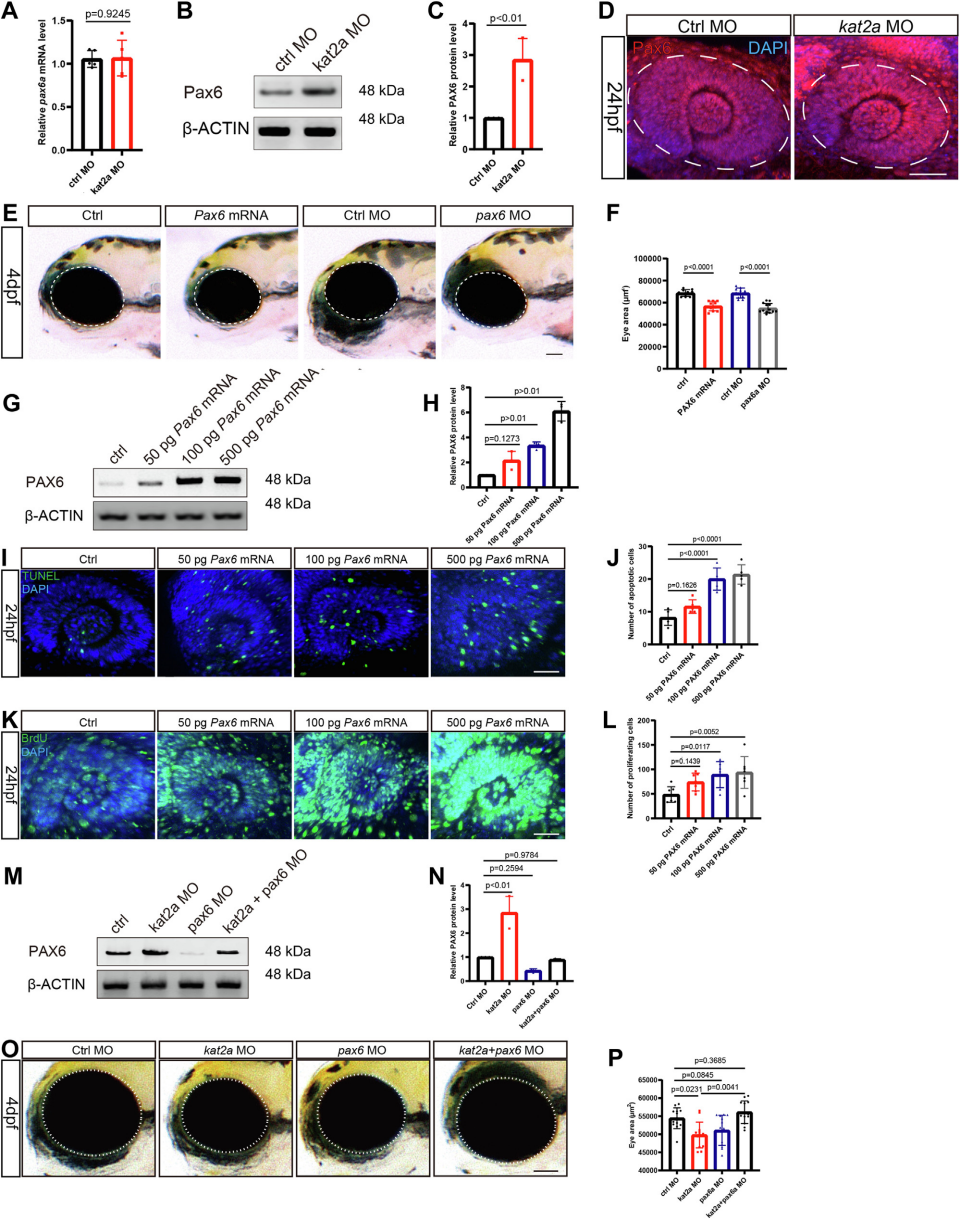
**Downregulation of *kat2a* reduced Pax6 and increased apoptosis.***A*, mRNA level of *pax6a* was unchanged in the eyes of 0.3 mM *kat2a* MO zebrafish morphants, compared with siblings that received control MO injection. *B* and *C*, protein level of Pax6 was increased in the eyes of 0.3 mM kat2a MO zebrafish morphants. n = 3, Student’s *t* test. Data were normalized to control MO, which was considered as one. *D*, immunohistological staining of Pax6 in 0.3 mM *kat2a* MO zebrafish morphants showed increased Pax6 level compared with control morphants. The scale bar represents 50 μm. *E*, morphology of zebrafish with overexpressed Pax6 or downregulated Pax6. The scale bar represents 50 μm. *F*, statistical diagram of eye sizes of zebrafish with overexpressed PAX6 or downregulated Pax6, n = 10, 10, 10, 13 zebrafish embryos, Student’s *t* test. *G* and *H*, Western blotting of Pax6 level in zebrafish embryos injected with 0, 50, 100, or 500 pg *Pax6* mRNA. n = 3, 3, 3, 3 batches of injected zebrafish embryos, each batch containing 30 embryos. *I*–*L*, BrdU and TUNEL assays detecting proliferating and apoptotic cells in zebrafish at 24 hpf with altered PAX6 expression. The scale bar represents 40 μm. *I*, n = 5, 5, 5, 5, one-way ANOVA followed by Dunnett’s multiple comparison. *L*, n = 7, 7, 7, 7, one-way ANOVA followed by Dunnett’s multiple comparison. *M* and *N*, Western blotting of Pax6 level in zebrafish embryos injected with control MO, *kat2a* MO, *pax6* MO, or mixed *kat2a* and *pax6* MO. n = 3, 3, 3, 3 batches of injected zebrafish embryos, each batch containing 30 embryos, two-way ANOVA followed by Fisher’s LSD. *O* and *P*), pax6a MO rescues the reduction of eye size in *kat2a* morphants. The scale bar represents 50 μm. n = 13, 13, 13, 13, two-way ANOVA followed by Fisher’s LSD. Control embryos for *Pax6* mRNA received mCherry mRNA injection, and control embryos for *pax6* MO received control MO injection. MO, morpholino oligonucleotides; TUNEL, terminal deoxynucleotidyl transferase-mediated dUTP nick-end labeling.

*kat2a* morphants caused a phenotype of small eyes. Owing to the similarity of the phenotype of *kat2a* knockout/knockdown and that of *pax6* mutations (27, 28, 29, 30), we questioned whether *pax6* is involved in the phenotype of *kat2a* morphants. The *pax6a* gene encodes PAX6 protein of zebrafish. We examined the level of Pax6 protein in the developing eye using immunofluorescence and Western blotting. The amount of Pax6 protein was increased in *kat2a* mutants compared with the wildtype siblings, while the mRNA level of *pax6a* was not significantly affected (Fig. 2, *A*–*D*).

To find out if an increase in Pax6 level was part of the reason for reduced eye size, we overexpressed *pax6a* in zebrafish embryos. The results showed that upregulation of *pax6a* was sufficient to cause a reduction in eye size that was similar to the phenotype of Pax6 MO (Fig. 2, *E* and *F*), and the higher dosage of Pax6 led to a severer abnormality in the eyes. We thus detected cell proliferation and apoptosis in developing eyes. The forced expression of PAX6 at doses from 50 pg to 500 pg were verified by Western blotting, and PAX6 overexpression caused accelerated proliferation and increased apoptosis (Fig. 2, *G*–*L*). Moreover, we downregulated *pax6a* using MO for comparison purposes, and the individuals with downregulation of *pax6a* exhibited small eyes too (Fig. S1, *A*–*D*). This suggests that Pax6 is essential for the expansion of NSCs in developing eyes. Similar to those with *kat2a* knockdown, embryos with excessive Pax6 had more apoptotic cells.

Kat2a harbors more function than just acetylating Pax6, but if the regulation of NSC differentiation by Kat2a is mainly mediated by the reduction of Pax6, we could rescue the effect of *kat2a* knockdown by downregulating Pax6 at the same time. We then performed a double knockdown of *kat2a* and *pax6a* in zebrafish embryos using morpholino oligonucleotides (0.3 mmol *kat2a* MO, 0.01 mmol *pax6a* MO) and observed the morphology of the eyes. The results showed that kat2a MO increased protein level of PAX6, which could be rescued by pax6a MO (Fig. 2, *M* and *N*). Although solely downregulating *kat2a* using 0.3 mmol MO results in eye malformation, 0.01 mmol *pax6a* MO rescued the eye phenotype in *kat2a* morphants (Fig. 2, *O* and *P*). These results strongly implicated that the small eye phenotype of *kat2a* MO was due to the accumulation of Pax6 protein in the developing eyes of zebrafish.

### Downregulation or inhibition of Kat2a results in elevated Pax6 levels and hampers the differentiation of cultured NSCs

**Figure 3:**
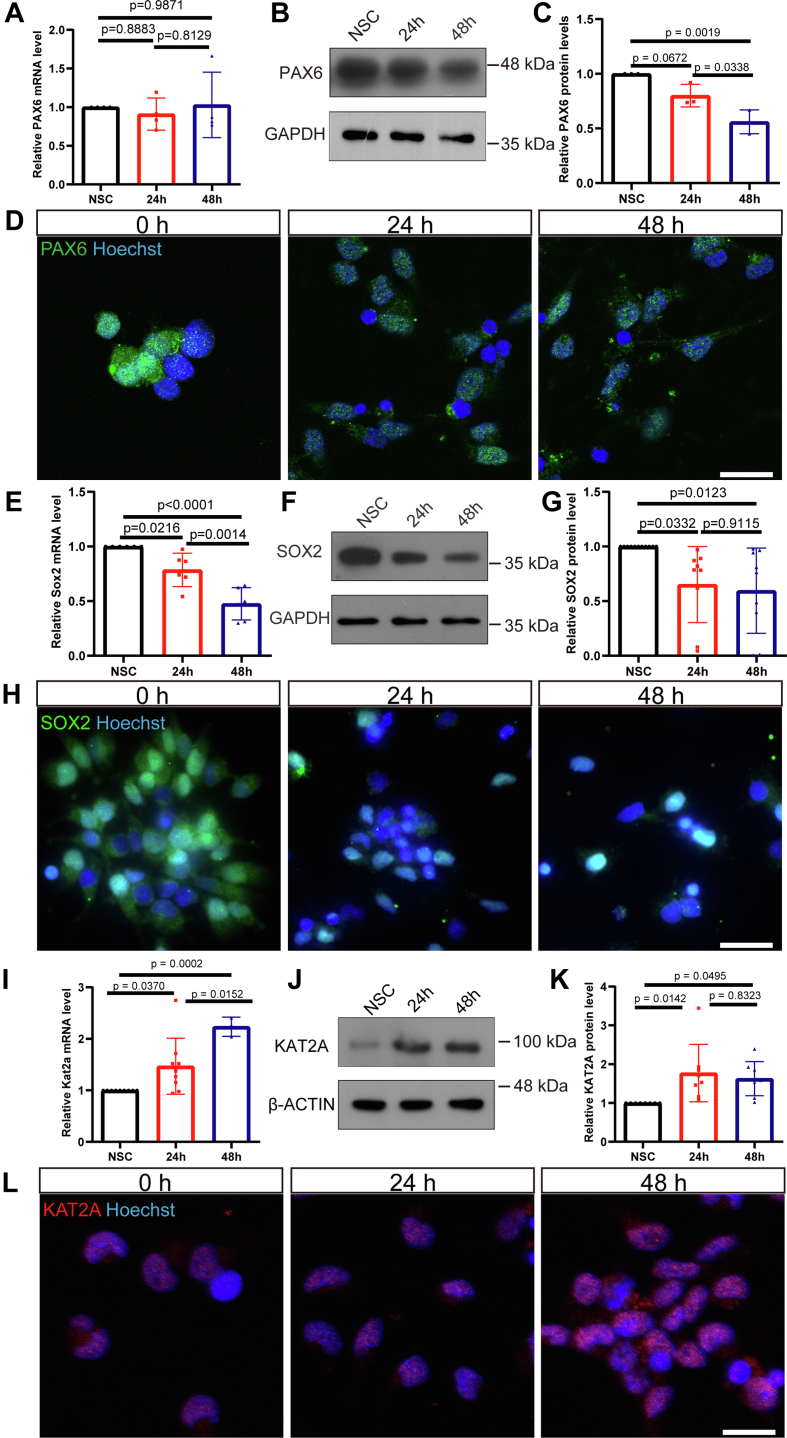
**PAX6 expression in differentiating neural stem cells.***A*, relative *Pax6* mRNA level did not vary significantly in differentiating NSCs, examined at 0 h, 24 h, and 48 h post induction. n = 4, 4 and 4 dishes of cultured NSCs, one-way ANOVA followed by Tukey’s multiple comparisons test. *B* and *C*, a decrease in PAX6 level detected by Western blotting in differentiating NSCs at 0 h, 24 h, and 48 h post induction. n = 3, 3 and 3 dishes of cultured NSCs, one-way ANOVA followed by Tukey’s multiple comparisons test. *D*, expression of PAX6 decreased in differentiating NSCs, examined at 0 h, 24 h, and 48 h post induction. The scale bar represents 20 μm. *E*, relative Sox2 mRNA level in differentiating NSCs at 0 h, 24 h, and 48 h post induction. n = 6, 6 and 6 dishes of cultured NSCs, one-way ANOVA followed by Tukey’s multiple comparisons test. *F* and *G*, SOX2 level detected by Western blotting in differentiating NSCs at 0 h, 24 h, and 48 h post induction. n = 11, 9 and 9 dishes of cultured NSCs, one-way ANOVA followed by Tukey’s multiple comparisons test. *H*, expression of SOX2 in differentiating NSCs, examined at 0 h, 24 h, and 48 h post induction. The scale bar represents 20 μm. *I*, relative Kat2a mRNA level in differentiating NSCs at 0 h, 24 h, and 48 h post induction. n = 9, 9 and 3 dishes of cultured NSCs, one-way ANOVA followed by Tukey’s multiple comparisons test. *J* and *K*, KAT2A level detected by Western blotting in differentiating NSCs at 0 h, 24 h, and 48 h post induction. n = 8, 8 and 8 dishes of cultured NSCs, one-way ANOVA followed by Tukey’s multiple comparisons test. *L*, expression of KAT2A in differentiating NSCs, examined at 0 h, 24 h, and 48 h post induction. The scale bar represents 20 μm. Data were normalized to NSCs at 0 h, which was considered as one. NSC, neural stem cell.

To confirm the relationship between KAT2A and PAX6 during neural development, we investigated the expression pattern of KAT2A and PAX6 in primarily cultured and differentiated NSCs from E14 rat embryos. *In vitro* differentiation of NSCs was induced using fibronectin and removal of FGF and EGF in the medium. PAX6 and SOX2 are transcription factors tightly related to neurogenesis. The RT-qPCR assay showed that mRNA of Pax6 did not vary significantly; however, protein PAX6 levels reduced along with differentiation that was verified by Western blotting and immunostaining assay (Fig. 3, *A*–*D*). Meanwhile, another transcription factor, SOX2, also showed decreased levels (Fig. 3, *E*–*H*). In contrast, the KAT2A level increased gradually during differentiation (Fig. 3, *I*–*L*). These data were consistent with the negative correlation between the levels of KAT2A and PAX6 in the developing eyes.

**Figure 4:**
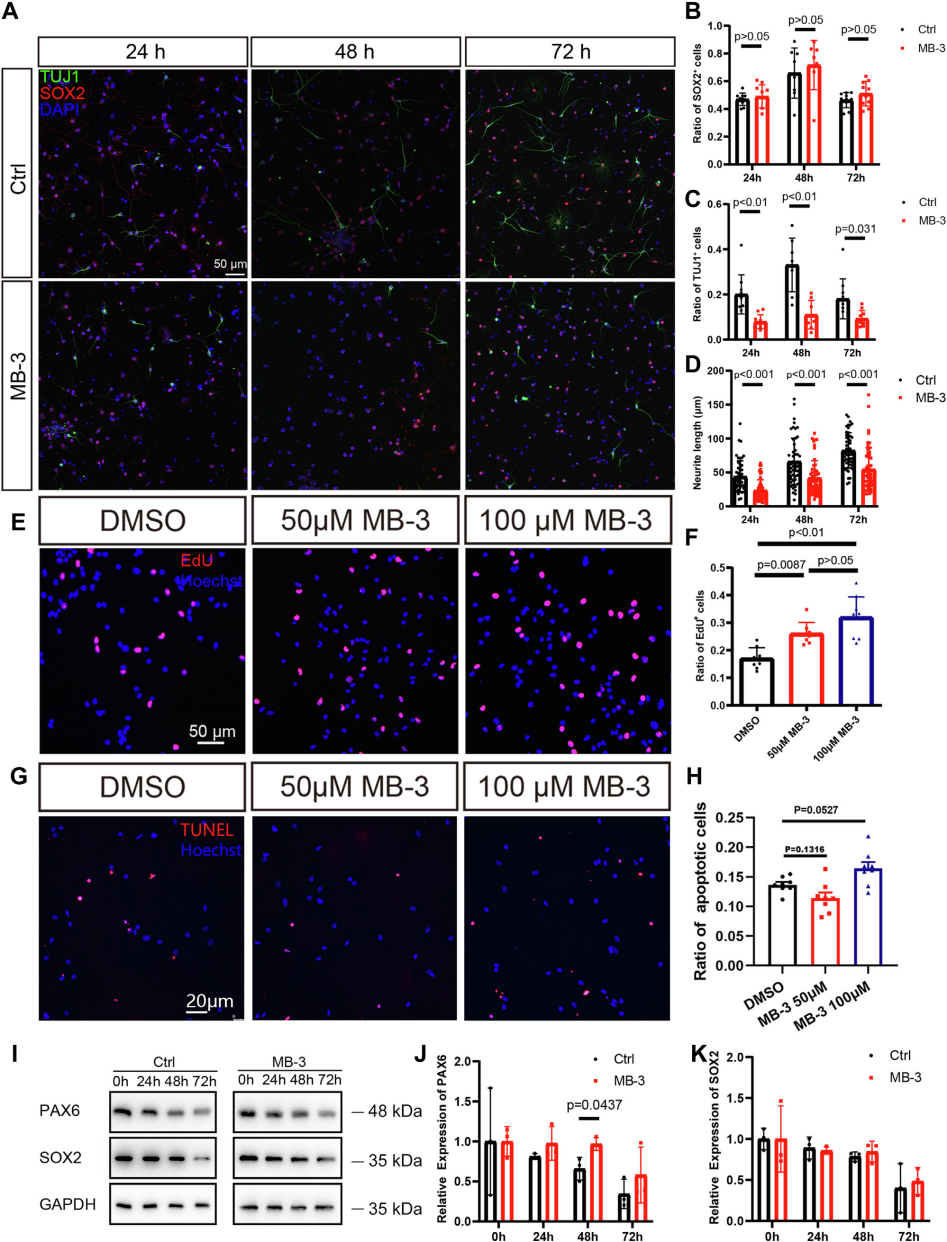
**Differentiation and PAX6 expression in differentiating NSCs treated with KAT2A-specific inhibitor MB-3.***A*–*D*, representative images and statistical diagram of ratio of SOX+ cells, ratio of TUJ1+ cells, reduced process length of differentiating NSCs treated with MB-3 for 24 h, 48 h, and 72 h, respectively. The scale bar represents 50 μm. n = 10, 10, 8, 8, 10, 10 dishes cells were imaged for SOX2- and TUJ1-expressing cells (five areas were selected for counting for each dish), and the ratio of SOX2- orTUJ1-expressing cells was obtained. Similarly, cells of each treatment group for neurite length were evaluated. Student’s *t* test. *E* and *F*, proliferation of NSCs was increased after MB-3 treatment, examined by immunofluorescence. The scale bar represents 50 μm. n = 8, 8, 9 dishes of NSCs. One-way ANOVA followed by Tukey’s multiple comparison tests. *G* and *H*, apoptosis of NSCs did not vary significantly after MB-3 treatment, examined by TUNEL. The scale bar represents 20 μm. n = 8, 8, 8 dishes of NSCs. One-way ANOVA followed by Tukey’s multiple comparison tests. *I*, PAX6 decreased during NSC differentiation but remained relatively stable with MB-3 treatment, examined by Western blotting. *J* and *K*, statistical diagrams of PAX6 and SOX2 protein level detected by Western blotting in differentiating NSCs treated with KAT2A-specific inhibitor MB-3. n = 3 dishes or cultured NSCs for each time point. Data were normalized to NSCs at 0 h. Two-way ANOVA followed by Fisher’s LSD was performed to analyze significance of differences. NSC, neural stem cell; TUNEL, terminal deoxynucleotidyl transferase-mediated dUTP nick-end labeling.

As PAX6 and KAT2A are negatively related in their protein levels during NSC differentiation, there could be a direct or indirect connection between the decrease of PAX6 and the activity of KAT2A in differentiating NSCs. First, we examined if the change in the level of PAX6 is in response to KAT2A. We applied KAT2A specific inhibitor MB-3 (31) to confirm if the change in PAX6 is related to the activity of KAT2A. After inhibition of KAT2A, the processes protruding from the differentiating cells were shorter than those of the control cells, and the percentage of TUJ1-positive cells suggested that differentiation was hindered (Fig. 4, *A*–*D*). The proliferation of NSCs increased upon treatment, while the apoptosis did not vary significantly (Fig. 4, *E*–*H*) at the dosages tested, which might be due to the sufficient supply of nutrients and space in the culture system. Then, we investigated whether the MB-3 treatment could affect the level of PAX6. As expected, in comparison with the control where PAX6 gradually declined along with differentiation, the expression of PAX6 remained steady throughout the 48 h of induction of differentiation (Fig. 4, *I* and *J*). In contrast, the SOX2 level declined during NSCs differentiation with statistical significance at 48 h and 72 h after induction of differentiation, and the treatment of MB-3 did not hamper the decline of SOX2 level (Fig. 4, *I* and *K*). Hence, we propose that the reduction of PAX6 level during NSC differentiation is specially regulated by KAT2A.

Also, we transfected NSCs with siRNA targeting *Kat2a* to downregulate the expression of KAT2A by electroporation and observed the NSCs after induction of differentiation for morphology of the cells, proliferation, and expression of PAX6. After 24 h of induction, the cells showed shorter processes, suggesting differentiation was inhibited after depletion of KAT2A (Fig. S2, *A* and *B*). We used EdU to label proliferating cells and found more proliferating cells after the knockdown of Kat2a (Fig. S2, *C* and *D*). Western blotting of PAX6 showed an elevated level of PAX6 after knockdown of Kat2a (Fig. S2, *E*–*G*). These results suggest that KAT2A downregulates the level of PAX6 and promotes differentiation in NSCs.

### KAT2A and PAX6 colocalize and interact in cultured NSCs

**Figure 5:**
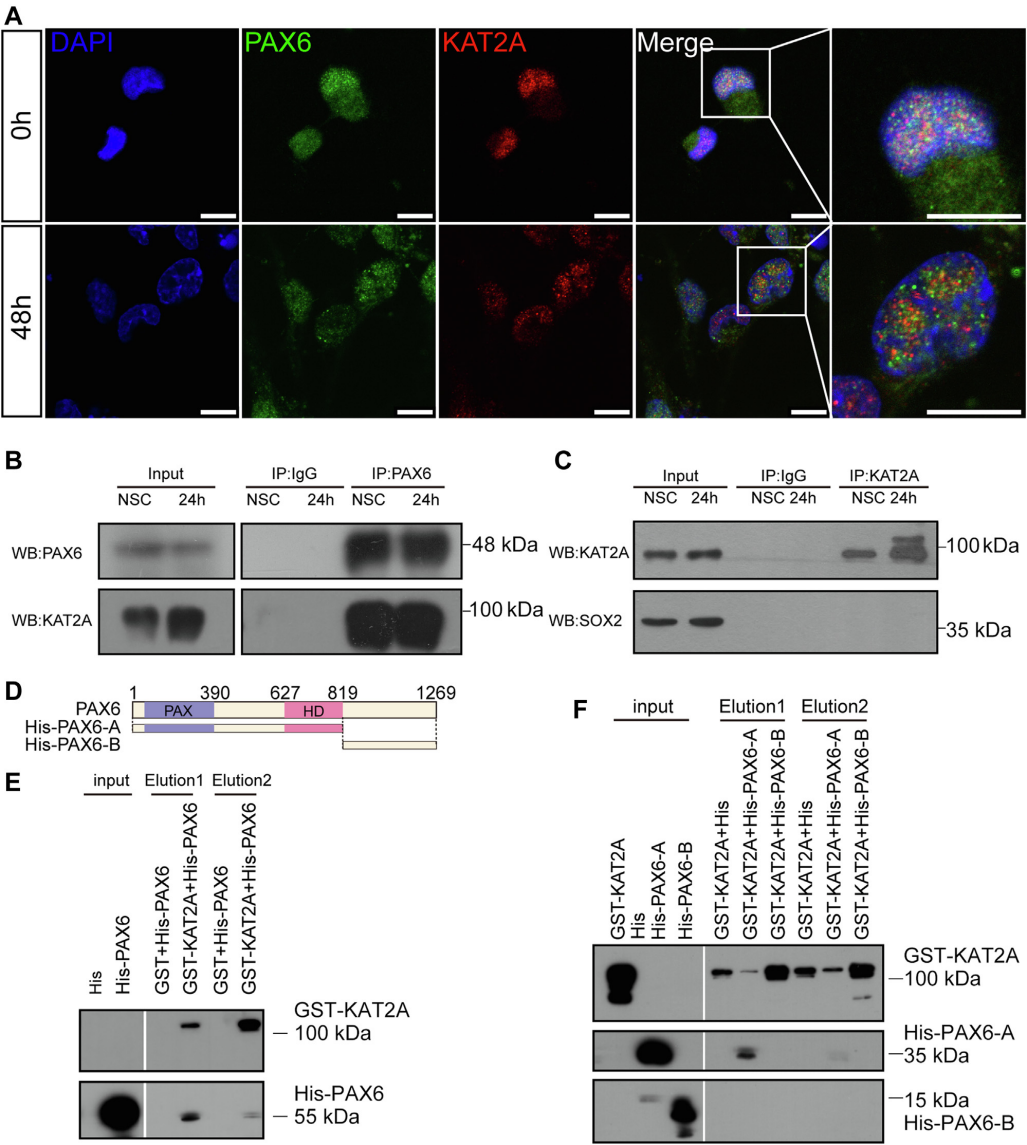
**Interaction between KAT2A and PAX6 followed by ubiquitination and degradation of PAX6.***A*, immunofluorescence staining in differentiating NSCs showing partial colocalization of KAT2A and PAX6. The scale bar represents 10 μm. *B*, interaction of KAT2A and PAX6 shown by immunoprecipitation of PAX6 followed by Western blotting of KAT2A. *C*, KAT2A does not interact with SOX2, as shown by immunoprecipitation of KAT2A followed by Western blotting of SOX2. *D*, diagram showing PAX6 truncated proteins used in this study. *E* and *F*, detection of the interaction between KAT2A and PAX6 using immunoprecipitation and Western blotting. KAT2A binds to PAX6 (*E*) in the N-terminal region from the PD domain to the HD domain and not the C-terminal region (*F*). NSC, neural stem cell.

KAT2A negatively regulates PAX6 level in NSCs; we then asked what the underlying mechanism is. The first question we addressed is whether the two molecules colocalize or interact in NSCs. We performed immunofluorescence staining on cultured differentiation NSCs to detect any interaction between KAT2A and PAX6. We detected colocalization of KAT2A and PAX6 both in undifferentiated and differentiated NSCs (Fig. 5*A*), with Pearson’s R values of 0.68 and 0.41, respectively. Meanwhile, the separate signals were also observed in NSCs. We believed that the KAT2A protein is multifunctional, and then both colocalized and separate signals with PAX6 protein could be present in cells.

Next, we performed an immunoprecipitation assay to find evidence for any interaction between KAT2A and PAX6. Using an anti-PAX6 antibody for immunoprecipitation, both Pax6 and KAT2A could be detected in the precipitate by Western blot (Fig. 5*B*). Meanwhile, the interaction between KAT2A and SOX2 was not detected (Fig. 5*C*) in NSCs. We also detected the expression of other HAT during NSCs differentiation. The results showed that CREB-binding protein (CBP) maintained a stable expression, and P300/CBP-associated factor (PCAF) displayed low expression level in NSCs, which was different from the pattern of KAT2A (Fig. S3, *A*–*D*).

To investigate whether KAT2A and PAX6 could interact directly, we conducted a GST-pulldown assay. The results showed that the GST-tagged KAT2A could successfully pulldown the His-tagged PAX6. We further generated several PAX6 truncated proteins and performed a GST-pulldown assay to locate the specific domain of PAX6 responsible to interact with KAT2A. We revealed that the N-terminal region containing the PD and HD domains is essential for the interaction (Fig. 5, *D*–*F*). Then, we dissected the N-terminal region into smaller truncated proteins and performed GST-pulldown for each of them and did not observe an interaction between these truncates and KAT2A (Fig. S4, *A*–*E*). These results suggested that the N-terminal region from the PD domain to the HD domain is required for the interaction between PAX6 and KAT2A.

### KAT2A acetylates PAX6 and facilitates ubiquitination of PAX6

**Figure 6:**
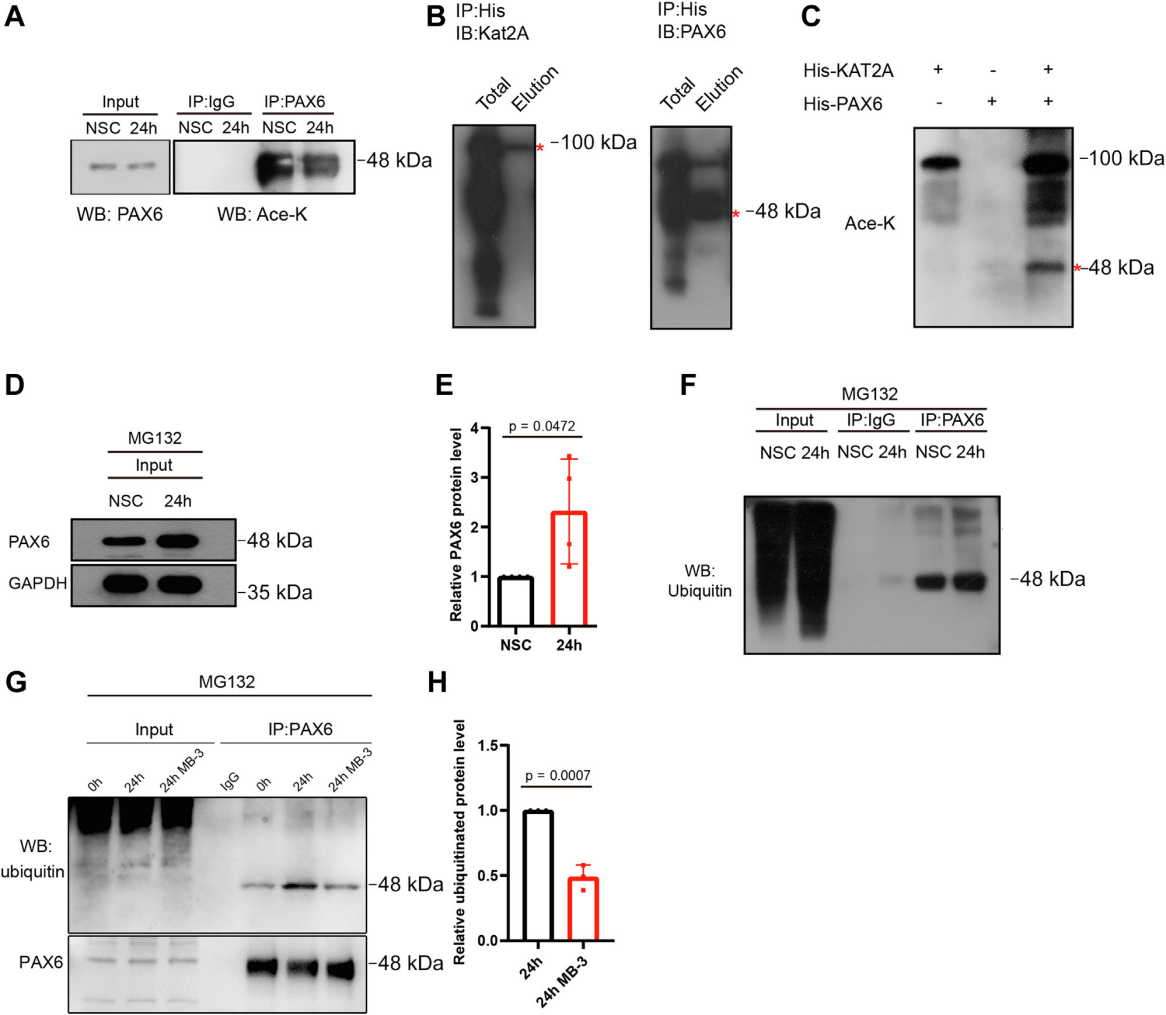
**Ubiquitination of PAX6 after inhibition of KAT2A in NSCs.***A*, acetylation of PAX6 was detected by Western blotting of acetylated lysine following immunoprecipitation of PAX6. *B*, purified His-KAT2A protein (*red asterisk*) and His-PAX6 protein (*red asterisk*). *C*, acetylated PAX6 (*red asterisk*) after interaction with KAT2A. *D* and *E*, expression of PAX6 detected in differentiating NSCs treated with 20 μM MG132 for 8 h after inhibition of proteasome using MG132 retains PAX6. n = 6 and 6 dishes of cultured NSCs. Student’s *t* test. Data were normalized to NSC at 0 h, which was considered as one. *F*, ubiquitinated PAX6 detected in differentiating NSCs treated with 20 μM MG132 for 8 h. *G* and *H*, ubiquitination of PAX6 was reduced after inhibition of KAT2A using MB-3 in differentiating NSCs. NSC, neural stem cell.

KAT2A is known for its acetyltransferase activity. To verify whether PAX6 is a substrate of KAT2A, we observed acetylated lysine in the precipitate obtained using PAX6 antibody from NSCs, suggesting lysine acetylation indeed occurred in NSCs (Fig. 6*A*). Also, we purified the His-tagged KAT2A and PAX6 protein expressed in *Escherichia coli* and conducted *in vitro* acetylation assay. The results clearly showed that acetylated PAX6 was observed in the presence of KAT2A and acetyl-CoA (Fig. 6, *B* and *C*).

Since there was a decline in the PAX6 level, which might be mediated by ubiquitination, we examined situations of PAX6 ubiquitination in differentiated NSCs and investigated whether the inhibition of KAT2A could affect PAX6 ubiquitination. For the reason that ubiquitinated PAX6 will experience degradation by proteasome, we applied proteasome inhibitor MG-132 (32) to hamper the degradation, then evaluated the level of Pax6 ubiquitination. We first detected the effect of MG-132 on the PAX6 level in NSCs and found that the decline of PAX6 during differentiation was attenuated upon treatment, which indicates that proteasome-mediated degradation is responsible for the PAX6 decline during differentiation (Fig. 6, *D* and *E*). Then, we immunoprecipitated PAX6 in NSCs in the presence of MG-132 and detected the ubiquitinated PAX6 with an antibody against ubiquitinated lysine residues. The results showed that the ubiquitinated Pax6 could be detected both in undifferentiated and differentiated NSCs (Fig. 6*F*). Then, we questioned whether the ubiquitination of PAX6 was regulated by the acetylation of PAX6 by KAT2A. We thus inhibited the activity of KAT2A in NSCs by MB-3 and found the ubiquitination level of PAX6 decreased (Fig. 6, *G* and *H*). These results support that, along with NSC differentiation, PAX6 is ubiquitinated, which is promoted by its acetylation catalyzed by KAT2A.

### The identification of acetylation and ubiquitination site in PAX6

**Figure 7:**
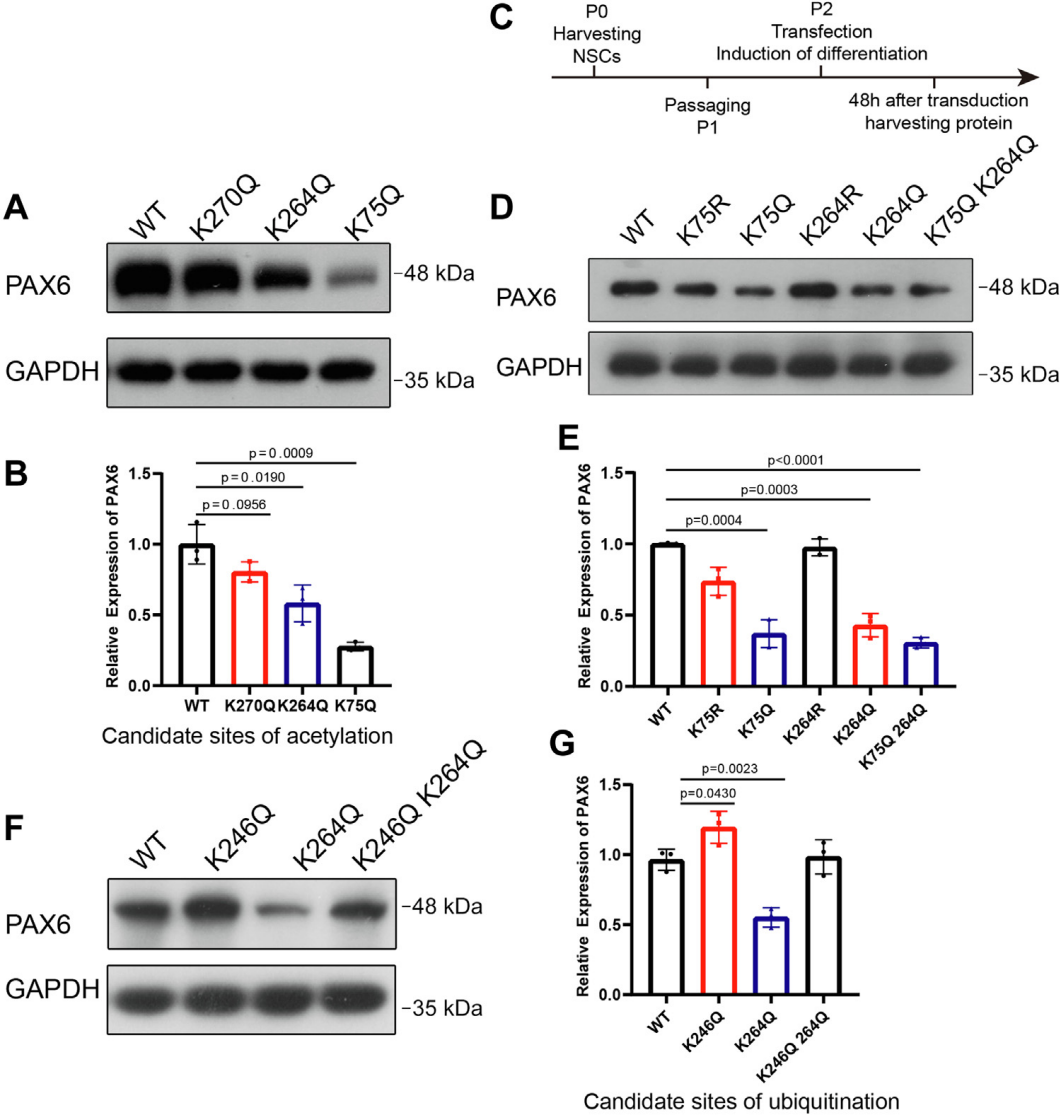
**Analysis of site of acetylation and ubiquitination in PAX6.***A* and *B*, K75 and K264 are the candidate site of acetylation in PAX6, detected in COS-7. n = 3 and 3 dishes of transfected cells, one-way ANOVA followed by Dunnett’s multiple comparisons test. *C*, time points of treatment and examination in NSCs. *D* and *E*, in cultured NSCs, PAX6 remained stable with K75R or K264R acetylation-defective mutation and was significantly decreased with K75Q and/or K264Q mutations mimicking acetylation. n = 3 and 3 dishes of transfected cells, one-way ANOVA followed by Dunnett’s multiple comparisons test. *F* and *G*, PAX6 K246Q was more stable in COS-7, suggesting K246 is not a site of acetylation. PAX6 with an acetylation-mimicking mutation K264Q underwent more degradation. An additional K246Q mutation blocks the increased degradation, suggesting K246 is a site of ubiquitination. n = 3 and 3 dishes of transfected cells, one-way ANOVA followed by Dunnett’s multiple comparisons test. Transfection of the plasmid coding wildtype PAX6 was used as control, and data were normalized to control. NSC, neural stem cell.

To determine the site of acetylation of PAX6, we conducted mass spectrometry analysis of PAX6 from the immunoprecipitates from NSCs, and the results proposed three potential lysine acetylation sites, K264, K75, and K270 of PAX6 (Fig. S5). To verify this, GFP-tagged PAX6 mutants in which each of the three lysine residues was changed to glutamine *via* site-directed mutagenesis to mimic the lysine acetylation were transfected into COS7 cells. Compared with WT PAX6, K75Q and K264Q showed markedly reduced PAX6 protein (Fig. 7, *A* and *B*). Meanwhile, the protein level of the K270Q mutant did not change significantly. We then confirmed the phenotype of K75Q and K264Q mutant in NSCs. In this assay, the double-mutant K75Q/K264Q was also tested, and the control mutants K75R and K264R were introduced as well. The results showed that the mutants K75Q, K264Q, and K75Q/K264Q led to a reduced level of PAX6, and the wildtype and K75R and K264R mutants did not show a similar effect (Fig. 7, *C*–*E*). Therefore, our data proposed that the acetylation of K75 and K264 contributed to PAX6 degradation.

Then, we further investigated the ubiquitination sites of PAX6 during NSCs differentiation. We did not obtain the information on ubiquitinated PAX6 in the data of mass spectrometry, which might be due to the instability of ubiquitinated proteins. We thus predicted the sites of ubiquitination for PAX6 using the online software BDM-PUB (http://bdmpub.biocuckoo.org/index.php), which indicates K246 as a potential candidate with the highest score. We designed the mutant K246Q to test the effect on the PAX6 level. The results showed that the mutation led to attenuation of Pax6 degradation, which proposed that K246 was the site of ubiquitination. Moreover, K264Q mutation resulted in augmented degradation of PAX6, while the double mutation K246Q/K264Q significantly rescued the effect of K264Q (Fig. 7, *F* and *G*). These results strongly indicated that K246 ubiquitination was a site of ubiquitination that might be regulated by acetylation modification of PAX6.

### Kat2a regulates PAX6 degradation *via* RNF8-mediated ubiquitination

**Figure 8:**
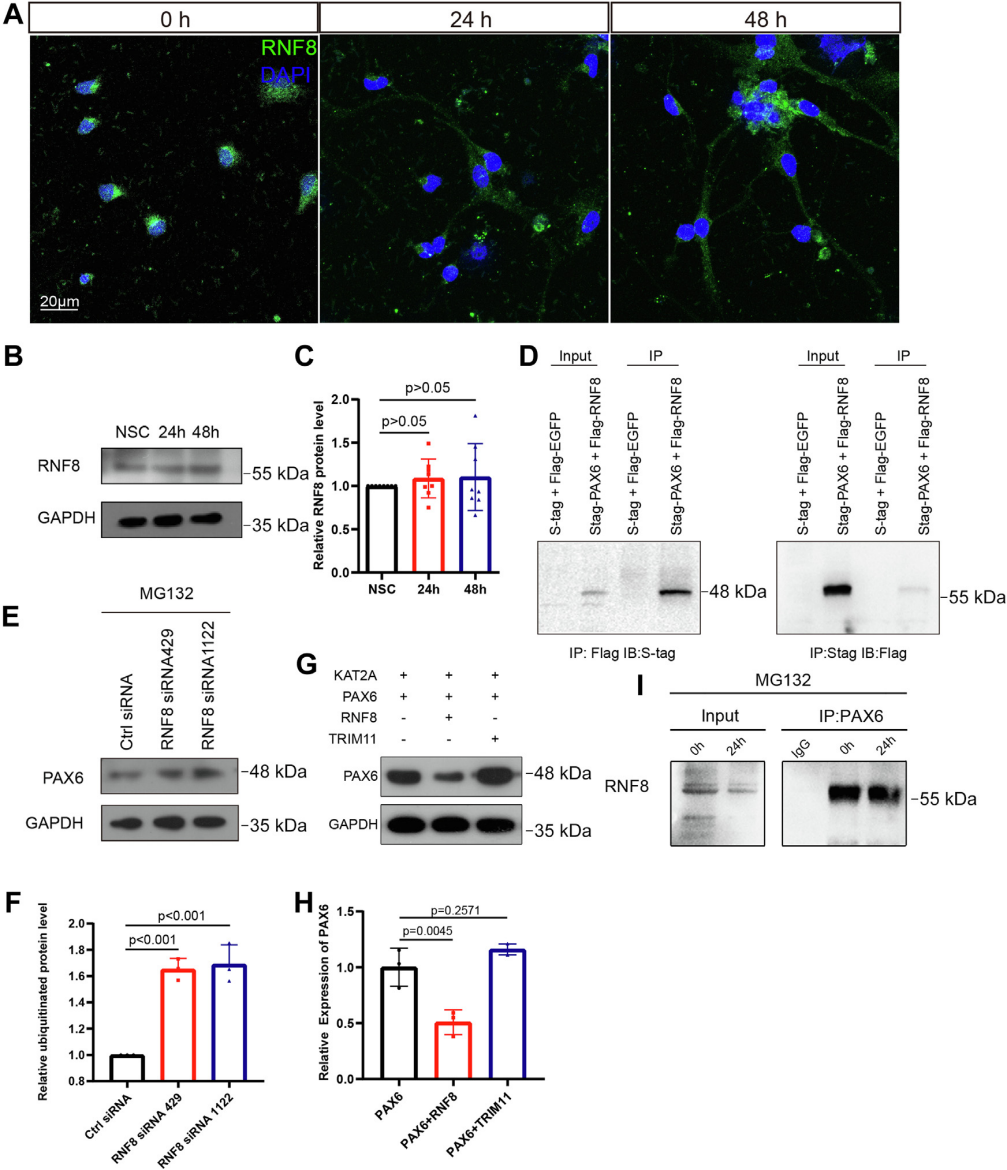
**Analysis of the E3 ligase responsible for the PAX6 ubiquitination.***A*, representative immunofluorescence images showing RNF8 expression at 0 h, 24 h, and 48 h after induction of differentiation. *B* and *C*, relative RNF8 protein level in differentiating NSCs determined by Western blotting. n = 8, one-way ANOVA followed by Dunnett’s multiple comparisons test. Data were normalized to NSC at 0 h, which was considered as one. *D*, RNF8 interacts with PAX6 in HEK293T, as shown by immunoblotting of PAX6 following immunoprecipitation of RNF8 and immunoblotting of RNF8 following immunoprecipitation of PAX6. *E* and *F*, depletion of RNF8 using siRNA retains PAX6 in NSCs increased PAX6 level, determined by Western blotting. n = 3, one-way ANOVA followed by Dunnett’s multiple comparisons test. Data were normalized to ctrl siRNA. *G* and *H*, RNA8 and not TRIM11 is the E3 ligase responsible for PAX6 ubiquitination, detected in COS-7 cells. n = 3 and 3 dishes of transfected cells, one-way ANOVA followed by Dunnett’s multiple comparisons test. Data were normalized to the “PAX6” transfection samples. *I*, RNF8 interacts with PAX6 at 0 h and 24 h after induction of NSCs differentiation that was detected by coimmunoprecipitation assay. NSC, neural stem cell.

To search for E3 ubiquitin ligases involved in the PAX6 degradation pathway, we retrieved the mass spectrometry results of the immunoprecipitation reaction of PAX6 from NSCs and found an E3 ligase RNF8, which is involved in DNA damage response in previous reports (33, 34). Then, we detected the expression of RNF8 in NSCs and found that RNF8 was fairly stably expressed during differentiation (Fig. 8, *A*–*C*). Moreover, we performed the coimmunoprecipitation assay in HEK293T cells that were transfected with the tagged RNF8 and PAX6. The results clearly confirmed the interaction of RNF8 and PAX6 (Fig. 8*D*). In addition, the endogenous RNF8 could be coimmunoprecipitated with PAX6 in cultured NSCs (Fig. 8*I*). Inhibition of RNF8 expression protected PAX6 protein from degradation (Fig. 8, *E* and *F*). Actually, another E3 ligase, Tripartite Motif Containing 11 (TRIM11) had been reported to play a role in the degradation of insoluble PAX6. Tuoc *et al.* (35) detected the interaction by the experiments in which HA-Trim11 plasmid was electroporated into primary cortical cultures prepared from E12.5 embryo brains. We also found the interaction between TRIM11 and PAX6 in our system (data not shown). Therefore, we could not ignore the potential effect of TRIM11 on PAX6. Then, we tested whether *Rnf8* or *Trim11* could reduce the PAX6 level. *Rnf8* or *Trim11* were transfected together with *Kat2a* and *Pax6*, respectively, into COS7 cells, and the results showed that the level of PAX6 protein decreased after transfection with *Rnf8* while not *Trim11* (Fig. 8, *G* and *H*), suggesting that RNF8 was the involved E3 ubiquitin ligase. The results above suggest that KAT2A promotes ubiquitination of PAX6 by RNF8 during the differentiation of NSCs.

**Figure 9:**
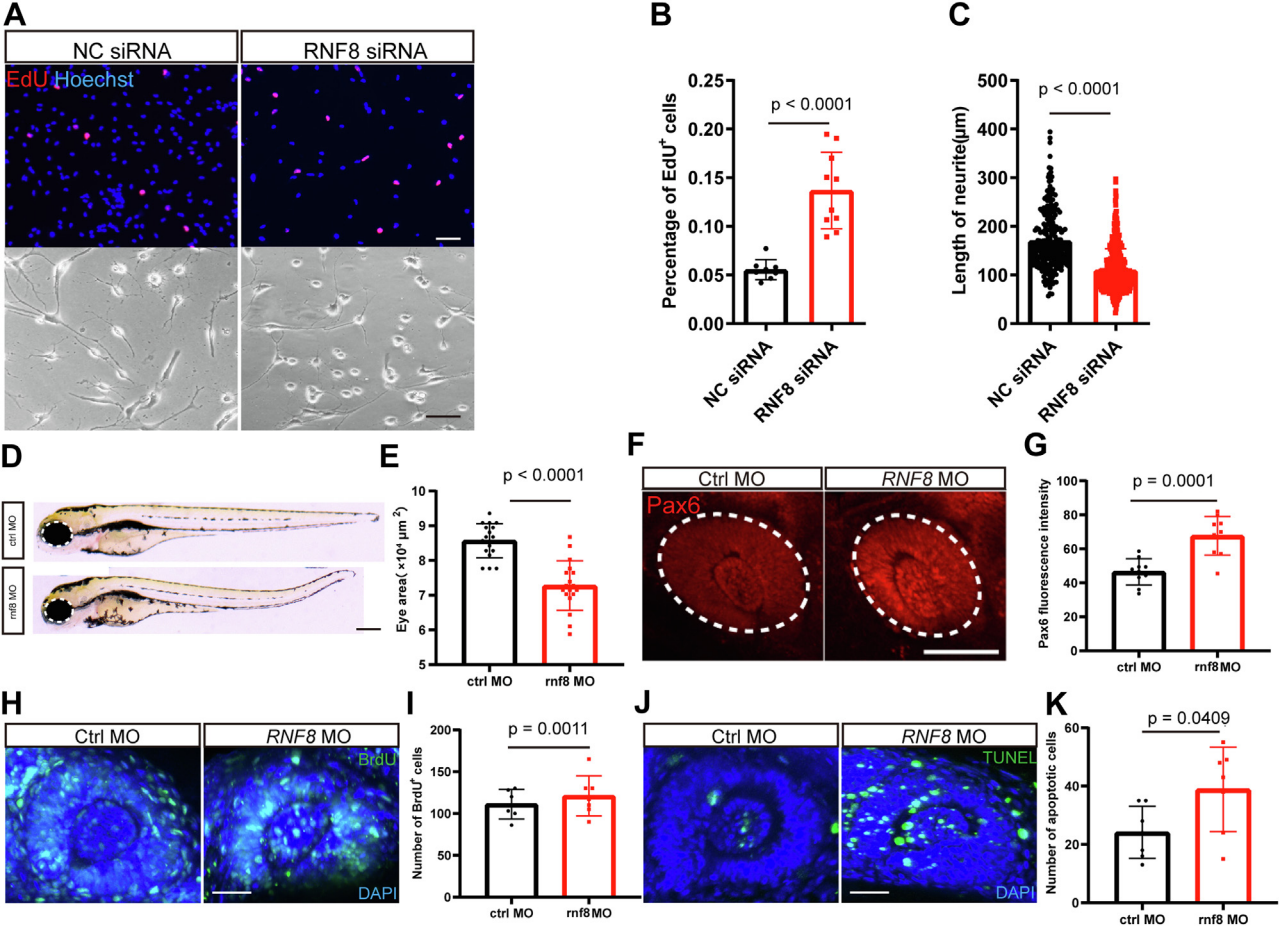
**Effect of loss of function of RNF8 in differentiating neural stem cells and developing zebrafish embryos.***A*–*C*, depletion of RNF8 in differentiating neural stem cells increases proliferation and inhibits differentiation, compared with cells transfected with negative control siRNA. The scale bar represents 50 μm. n = 8 and 10 for EdU proliferation analysis, n = 258 and 455 cells for neurite length measurement, Student’s *t* test. *D* and *E*, eye size was reduced in zebrafish rnf8 morphants compared with control morphants. The scale bar represents 200 μm. n = 18 and 18 zebrafish, Student’s *t* test. *F* and *G*, the expression of Pax6a was increased in the eyes of zebrafish *rnf8* morphants compared with control morphants. The scale bar represents 200 μm. n = 10 and 10 zebrafish, Student’s *t* test. *H* and *I*, the number of proliferating cells was increased in the eyes in zebrafish rnf8 morphants compared with control morphants. The scale bar represents 50 μm. n = 6 and 7 zebrafish, Student’s *t* test. *J* and *K*, the number of apoptotic cells was increased in the eyes in zebrafish rnf8 morphants compared with control morphants. The scale bar represents 50 μm. n = 7 and 7 zebrafish, Student’s *t* test.

To confirm that RNF8 is an essential player in the regulation, we observed the effect of RNF8 inhibition in differentiating NSCs and developing zebrafish embryos. Effective downregulation of RNF8 appeared to cause a mimic phenotype of downregulation or inhibition of KAT2A in NSCs, in terms of promoting proliferation and reducing the length of neurites in neuron-like cells (Fig. 9, *A*–*C*). In developing zebrafish embryos, the downregulation of *rnf8* reduced eye sizes, promoted the accumulation of Pax6, and increased both proliferation and apoptosis (Fig. 9, *D*–*K*), which showed similar phenotype as the embryos with downregulation of kat2a. These data also support the idea that RNF8 participates in the PAX6 degradation that is facilitated by the KAT2A-mediated acetylation.

**Figure 10:**
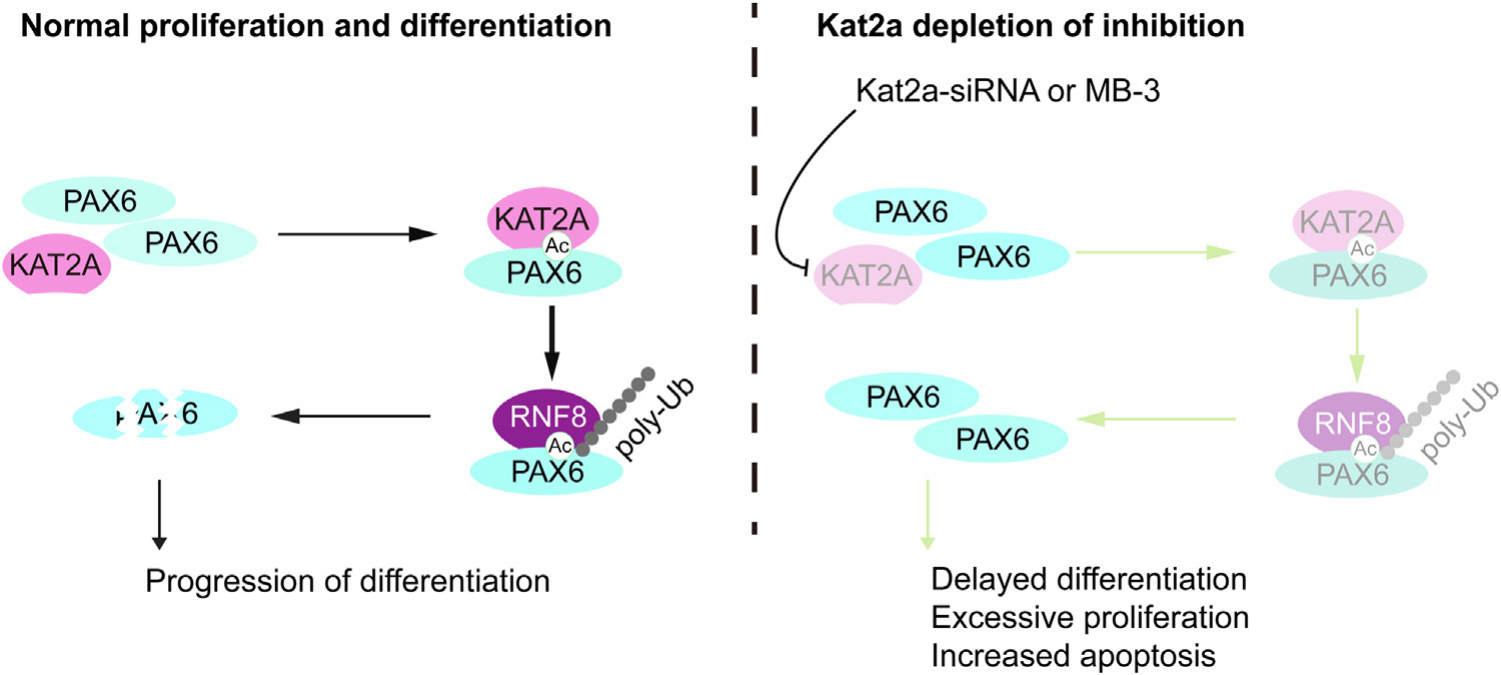
**Working model of KAT2A acetylates PAX6 and promotes its ubiquitination to regulate homeostasis and behavior of NSCs.** With normally regulated KAT2A, PAX6 is acetylated and then ubiquitinated and degraded to promote the differentiation of NSCs. When KAT2A is depleted or inhibited, acetylation and ubiquitination of PAX6 are obstructed and the accumulation of PAX6 blocks normal NSC differentiation. NSC, neural stem cell.

In summary, we revealed that KAT2A binds to and acetylates PAX6 to promote its ubiquitination mediated by RNF8, which in turn intensifies the downregulation of PAX6 to inhibit the proliferation and drive the differentiation of NSCs. Upon knockdown or inhibition of KAT2A, a decrease in KAT2A weakens the KAT2A-dependent degradation of PAX6, and the accumulated PAX6 leads to overproliferation and apoptosis (Fig. 10). This study discovered a novel pathway that might play an important role in NSCs homeostasis during development.

## Discussion

Transcription factors modification is essential for their transcriptional activity, distribution, stability, and degradation. In this study, we revealed a KAT2A/PAX6/RNF8 axis that controls the level of PAX6 during NSCs differentiation. We found that KAT2A-mediated acetylation of PAX6 leads to enhanced degradation of PAX6, which modulated the proliferation, differentiation, and apoptosis of NSCs.

KAT2A is the first reported histone acetyltransferase that links histone acetylation to transcriptional regulation of the target gene, and its role in transcription activation has been extensively investigated (36, 37). Recent studies showed that KAT2A-mediated acetylation was involved in Cyclin A turnover of *Drosophila* germline stem cells (38), which proposed a novel mechanism of KAT2A-regulated protein degradation. In our study, we found that KAT2A mediated PAX6 reduction in a transcription-independent manner. The mRNA levels of PAX6 did not respond to KAT2A inhibition in developing eyes of zebrafish and *in vitro* cultured NSCs, while the degradation of PAX6 was halted upon treatment of KAT2A inhibition. Moreover, we verified that KAT2A interacted specially with PAX6 in NSCs, while another transcription factor Sox2 was not involved. These data showed that the KAT2A/PAX6 axis played an important role during NSCs differentiation. Toma-Fukai *et al.* (39) reported that KAT2A also contains an atypical ubiquitin ligase structure and exhibits ubiquitination activity. This issue raises the question whether KAT2A could directly facilitate PAX6 ubiquitination. In our study, we did not detect the ubiquitination activity of KAT2A directly, while we demonstrated that the E3 ligase, RNF8, significantly enhanced PAX6 degradation. Meanwhile, the inhibition of RNF8 showed a similar phenotype as KAT2A inhibition in NSCs. These data implied that KAT2A-mediated PAX6 degradation is dependent on RNF8.

PAX6 homeostasis is critical for the fate determination of NSCs, and the function of PAX6 has been extensively investigated. However, the reported effects of PAX6 on NSCs are still controversial (18, 19, 40). The possible reason might be that the effect of PAX6 is context dependent, and the dosage of PAX6 is also critical for its function, which proposed that the refined orchestration of PAX6 is essential for its proper function. In this study, we demonstrated that the low pax6 level resulted in insufficient proliferation of NSCs in the developing eyes of zebrafish. These data are consistent with the function of PAX6 in the proliferation and expansion of retinal stem cells in mice (18). We further found enhanced expression of PAX6 significantly promoted cell proliferation and apoptosis. We tried a series of dosages and found that cell proliferation is constantly increased even the developing eyes showed severe deformity. The results are controversial to the report on developing cerebral cortex of mice when PAX6 levels were elevated (20, 21). These data implied that PAX6 might play a differential role in proliferation in the eyes than in the cerebral cortex during development.

We demonstrated that KAT2A acetylated PAX6 and facilitated the ubiquitination of PAX6 by RNF8. Tuoc *et al.* reported that PAX6 interacted with another E3 ligase Trim11using GST-pulldown assay and the inhibition of TRIM11 led to evaluated PAX6 level during cerebral cortex development in mice. However, we found RNF8 promoted PAX6 degradation more efficiently than TRIM11 in cultured NSCs or COS7 transfected simultaneously with constructs of PAX6 and RNF8 or TRIM11. These results sound paradoxical to the previous study of PAX6 and TRIM11. We noted that Tuoc *et al.* showed the effect of TRIM11on insoluble PAX6, which implied that TRIM11 might be responsible for the turnover of the PAX6 protein, and RNF8 was more likely involved in an active regulation of PAX6 upon differentiation stimulus. In addition, we noticed that there is no orthologue of the mammalian TRIM11 in zebrafish, but there is a 1-to-1 orthologue of mammalian RNF8. Our results suggest the KAT2A–RNF8–PAX6 regulation loop is conserved in vertebrates.

It can be seen in our study that an excessive amount of PAX6 promotes apoptosis, while an insufficient amount of PAX6 inhibits proliferation. Then we conclude from our data that a balanced amount of PAX6 is crucial for the proper development of the eye.

## Experimental procedures

### Animal husbandry and embryo injection

Zebrafish (Tübingen strain) were provided by the Zebrafish Facility at Nantong University under standard laboratory conditions of 14 h light/10 h darkness at 28.5 °C in E3 buffer, which contains 6.4 mM KCl, 0.22 mM NaCl, 0.33 mM CaCl_2_·2H2O, 0.33 mM MgSO_4_·7H2O in H2O.

Each embryo received about 1 nl of the Morpholino Oligonucleotide solution (sequences listed in Table 1, purchased from Gene Tools) using borosilicate glass capillaries with an IM-400 Electric Microinjector (Narishige). Control embryos received injections of an equal amount of control Morpholino Oligonucleotides provided by Gene Tools. The injected embryos were grown and harvested at 24 hpf for RNA isolation using TransZol Up Plus RNA Kit (TransGen Biotech) or observation at stated time points.

**Table 1:** Sequences of primers, siRNAs, and morpholino oligonucleotides used in this study

Primer/MO	Sequence (5′- 3′)
Standard control MO	cctcttacctcagttacaatttata
gcn5-sp-MO	aggtctgattcttcctacccaaaga
pax6a-sp-MO	acggagcacaggtattctcctcacc
rnf8-sp-MO	atttgcatggaattgctcacctcta
Control siRNA	s:uucuccgaacgugucacgutt as:acgugacacguucggagaatt
KAT2A-Rat-370 siRNA	s: gcuugcaaggccaaugaaatt as: uuucauuggccuugcaagctt
KAT2A-Rat-1383 siRNA	s: ccgagugaugggcgacauutt as: aaugucgcccaucacucggtt
KAT2A-Rat-1553 siRNA	s: gcaacucucugacgcccaatt as: uugggcgucagagaguugctt
PAX6 siRNA	s: gcagaagaucguagagcuatt as: uagcucuacgaucuucugctt
RNF8-Rat-429 siRNA	s: ggagcaugcggaguacgaatt as: uucguacuccgcaugcucctt
RNF8-Rat-1065 siRNA	s: ggguuuggagaaagagcaatt as: uugcucuuucuccaaaccctt
RNF8-Rat-1122 siRNA	s: gcaggagcaucgcgcucuatt as: uagagcgcgaugcuccugctt
Rat PAX6-F	cttggtgctgtctttgtca
Rat PAX6-R	atggagccagtctggtaat
Rat KAT2A-F	tcatcggtgggatttgct
Rat KAT2A-R	tactcgtcggcgtaggtg
Rat GAPDH-F	ccatcactgccactcagaagact
Rat GAPDH-R	acattgggggtaggaacacg

Sprague–Dawley rats were obtained from the Lab Animal Facility at Nantong University. All the studies reported here were submitted to Ethics Committee on Animal Experimentation of Nantong University, and all procedures were approved (S20220311-001) by the Animal Care and Use Committee of Nantong University.

### Immunofluorescence, BrdU staining, and TUNEL in zebrafish embryos

For immunofluorescence, 15 zebrafish embryos were pooled in a 1.5-ml tube and soaked using 1 ml 4% paraformaldehyde (PFA) (in PBS) at room temperature for 5 min and repeated with fresh 4% PFA for another 5 min. Then the sample was fixed in another 1 ml of 4% PFA for 30 min. The embryos were then washed with 1× PBST (contains 0.1% Tween-20) three times, 10 min each. Then the embryos were placed in 1× PBST (1% TritonX-100) at room temperature for 30 min. Next, the embryos were placed in 500 μl of blocking reagent (1 ml goat serum, 0.3 g bovine serum albumin, 100 μl Triton X-100, and 1x PBS to a final volume of 10 ml) for 2 h at room temperature. The embryos were incubated with 500 μl of 1:400 primary antibody solution at 4 °C for 4 days. The embryos were washed with 1× PBST (containing 0.1% Tween-20) four times, 10 min each. The embryos were incubated with 300 μl of secondary antibody solution (1:500) at 4 °C for 2 days. Again, the embryos were washed with 1× PBST (containing 0.1% Tween-20) four times, 10 min each. The embryos were stained with 500 μl of DAPI (10 μg/ml) for 1 h and finally washed with 1× PBST (containing 0.1% Tween-20) three times, 10 min each. Antibodies used were Rabbit-anti-PAX6 (1:100, 901301, BioLegend) and Cy3 AffiniPure Goat Anti-Rabbit IgG (H + L) (1:500, Jackson immunology 111-165-003).

For BrdU staining, the embryos were first placed on ice in E3 buffer for 15 min, and then changed with 1 ml 10 mM BrdU/15% dimethyl sulfoxide (DMSO) and kept on ice for 30 min. Then they were transferred into E3 buffer at 28.5 °C and kept at 28.5 °C for 5 min. Then they were fixed with PFA and transferred into methanol and kept at −20 °C overnight. The embryos were rehydrated in methanol: PBST (3:1), methanol: PBST (1:1), and methanol: PBST (1:3) in tandem, 5 min each, and washed with PBST twice, 5 min each. Then the embryos were digested with Proteinase K (10 μg/ml) for 10 min, washed with PBST for 10 min, and postfixed using PFA for 20 min. The embryos were washed with water quickly three times, in 2 mol/l HCl two times, and incubated in 2 mol/l HCl for 1 h, and washed in PBST three times, 5 min each. The samples were then soaked in blocking solution (0.2% blocking reagent, 10% fetal calf serum, 1% DMSO, 1xPBST) for 30 min and then incubated in 1:100 BrdU (Bu20a) Mouse mAb anti-BrdU antibody (CST #5292) at 4 °C for 5 days and washed five times in PBST, 10 min each. Next, the samples were incubated in 1:500 Alexa Fluor 488 AffiniPure Goat Anti-Mouse IgG (H + L) (1:500, Jackson immunology 115-545-003) at 4 °C for 2 days and washed in PBS five times, 10 min each. The embryos were finally stained with DAPI (10 μg/ml) for 1 h and washed with 1× PBS five times, 5 min each.

For the TUNEL experiments, the manufacturer’s instructions were followed (TUNEL BrightGreen Apoptosis Detection Kit, Vazyme).

### Cell culture and induction of differentiation

NSCs were isolated and induced for differentiation as reported (41, 42). Briefly, NSCs were prepared from the cerebral cortex of E14 Sprague–Dawley rats as described. The cells were maintained in DMEM/F12 medium (Cat. MA0214, Meilunbio) supplemented with 2% NeuroCult SM1 Neuronal Supplement (Cat. 05711, Stem Cell), 1% N2 Supplement-A (Cat. 07152, Stem Cell), 1% penicillin–streptomycin (Cat. C0222, Beyotime), 2 mM glutamine (Cat. C0212, Beyotime), 20 ng/ml recombinant bFGF (Cat. 450-33, Pepro Tech), and 20 ng/ml EGF (Cat. E9644, Sigma) on poly-l-lysine- (Sigma) and fibronectin- (Invitrogen) coated Petri dishes. EGF and bFGF were removed to induce NSC differentiation (43, 44, 45, 46, 47). Morphology of the cells was observed every 24 h during differentiation. From 24 h to 72 h after initiation of differentiation, the cells underwent significant morphological changes including the generation and elongation of the processes, suggesting that differentiation was successfully initiated.

COS7 cells were maintained and cultured in a DMEM medium (Cat. MA0212, Meilunbio) plus 10% FBS (Cat. S711-001S, Lonza). Cells were transfected using Lipofectamine 3000 (Cat. L3000015, Invitrogen) according to the supplier’s guidelines.

For MB-3 and MG132 treatments, the doses used were 50 μM for MB-3 (Sigma M2449) and 20 μM for MG132 (Selleck S2619).

### Immunofluorescence for cultured NSCs

The cultured cells were fixed in 4% paraformaldehyde for 20 min and were incubated with Rabbit anti-KAT2AL2 C26A10 (KAT2A) antibody (1:400, CST #3305), Mouse anti-KAT2A/KAT2A Monoclonal Antibody (1:400, LifeSpan LS-C133217), Rabbit anti-PAX6 antibody (1:400, BioLegend 901301), monoclonal Mouse anti-PAX6 antibody (1:400, Abcam ab78545), Rabbit anti-RNF8 antibody (1:400 Abcam ab105362), Rabbit anti-SOX2 antibody (1:400 Abcam ab97959), Rabbit anti-TUJ1 (1:400 Abcam ab18207), respectively. Fluorescence-labeled secondary antibodies (Alexa Fluor 488 affinipure Goat anti-rabbit IgG (H + L) [1:400, Jackson immunology 111-545-003], Cy3 affinipure Goat anti-rabbit IgG (H + L) [1:400, Jackson immunology 111-165-003], and Alexa Fluor 488 AffiniPure Goat Anti-Mouse IgG (H + L) [1:500, Jackson immunology 115-545-003]) were added and further incubated, followed by Hoechst 33342 staining. Visualization was performed on a Leica TCS SP5 confocal microscope.

### EdU cell proliferation detection

EdU Cell Proliferation Detection Kit (RiboBio) was used for cell proliferation detection. Briefly, EdU (Reagent A) was diluted into culture media at 1:1000 to a final volume of 50 mM, and cells were treated for 2 h before harvesting. Cells were then fixed with 4% PFA for 30 min and washed with 2 mg/ml glycine in PBS once and with PBS twice. Cells were then stained with 1× Apollo staining solution for 30 min and washed with PBS with 0.5% TritonX-100 for 10 min twice. Cells were stained with 1 μg/ml Hoechst 33342 for 15 min and washed with PBS for 5 min three times before Imaging.

### Western blotting

Western blot analysis was performed as described (48). Briefly, cells were lysed with RIPA buffer (Thermo Fisher Scientific), and obtained total proteins were quantified using a bicinchoninic acid assay (BCA) kit. After being subjected to SDS-PAGE, proteins were transferred onto a 0.2-μm PVDF membrane. After being blocked in 5% skim milk, the membranes were incubated overnight at 4 °C with primary antibodies. After washing with TBST (TBS with 0.1% Tween 20), horseradish peroxidase–conjugated secondary anti-mouse, rabbit, or rat antibodies were applied at room temperature for 2 h. The blot was covered with ECL solution (Cat.180-5001, Tanon) and visualized with X-ray film and then imaged using a scanner, and the data were analyzed using ImageJ software. GAPDH was used as a loading control.

Antibodies used were anti-PAX6 antibody (1:1000, Cat. Ab5790, Abcam), anti-KAT2A antibody (1:1000, Cat. Ab208097, Abcam), Mouse anti-β-Tubulin III (Tuj1) antibody (1:2000, BioLegend 801201), Mouse anti-GAPDH antibody (1:3000, Proteintech 60004-1-lg), Mouse anti-beta Actin antibody (1:2000, Proteintech 60008-1-lg), Rabbit anti-SOX2 antibody (1:2000, Abcam ab97959), Rabbit anti-CBP antibody (1:1000, CST D6C5), Mouse anti-PCAF antibody (1:1000, Santa Cruz sc-31324), Mouse anti-Ubiquitin antibody (1:1000, CST 3936), Rabbit anti-acetylated-lysine antibody (1:1000, CST 9441), HRP-conjugated Affinipure Goat Anti-Mouse IgG(H + L) (Proteintech, SA00001-1), and HRP-conjugated Goat anti-rabbit IgG (Santa Cruz sc-2054).

### Induced expression and purification of proteins

Expression plasmids were transformed into BL21 bacteria (TransGen). Expression was induced with 0.4 mM IPTG at 16 °C shaking at 120 rpm overnight. Proteins were quantified using the BCA method and purified with His or GST beads.

### GST-Pulldown

GST-pulldown was performed using Pierce GST Protein Interaction Pull-Down Kit (Pierce 21516) following the manufacturer’s instructions. Briefly, 50 μl GST beads were mixed with 125 μl TBS. 1 ml GST-bait protein was mixed with the beads and spun at 175 rpm for 30 min. The samples were placed on a magnetic stand for 10 s. The supernatant was collected and mixed with 6× loading buffer and boiled to form the postbinding control. The beads were washed with 250 μl TBS, mixed with a prey protein solution, and spun at room temperature at 175 rpm for 30 min. The beads were placed on the magnetic stand for 10 s. The supernatant was collected, mixed with 6× loading buffer, and boiled to form the post-IP control. The beads were washed with 250 μl TBS and spun at 175 rpm for 1 min and placed on the magnetic stand for 10 s to remove supernatant and washed repeatedly three times. A volume of 125 μl Elution Buffer was added to the beads for elution. A volume of 25 μl loading buffer was added to the elution before boiling for 10 min.

### Coimmunoprecipitation

Coimmunoprecipitation was performed using Pierce Co-Immunoprecipitation (Co-IP) Kit (Pierce 26149) following the manufacturer’s instructions. Briefly, 30 μl of S-protein Agarose was used for each reaction. Cells were treated with lysis buffer at 4 °C and centrifuged at 14,000*g* for 20 min. After measurement of protein concentration, part of the solution was mixed with 6× SDS loading buffer and boiled for 5 min and diluted to 2 μg/μl for the input control. The rest of the solution was diluted with PBS to 1.5 μg/μl, and 30 μl S-protein Agarose was added per 500 μl supernatant. The antibodies were then added to the samples and mixed by slow spinning at 4 °C overnight. The precipitate was collected by centrifuge at 14,000*g* for 15 min and washed with cool IP lysis buffer twice and resuspended with 60 μl 2× loading buffer. The samples were boiled for 8 min, briefly spun, and the supernatant was used for Western blotting.

### *In vitro* acetylation assay

A 50-μl reaction was prepared consisting of 5 μg PAX6, 1 μg KAT2A, 2 mM Ac-CoA, 50 mM Tris-HCl (pH 8.0), 100 mM EDTA, 1 mM PMSF, 5 mM DTT, 2.5 mM NAM, 1 μM TSA, and 10% glycerol. The reaction was placed at 37 °C for 2 h and stopped by adding 10 μl 6× loading buffer and incubating in boiling water for 5 min before detection with immunoblotting.

### mRNA preparation (PAX6 mRNA)

Full-length Pax6 cDNA was cloned by RT-PCR from a rat and inserted into pEasy-T3 (Transgen) and transcribed using mMessage mMachine Kit (Thermo Fisher Scientific).

### Real-time quantitative PCR

Reverse transcription was performed using a RevertAid First Strand cDNA Synthesis Kit (Thermo Fisher K1622), and PCR reactions were prepared using AceQ qPCR SYBR Green Master Mix (High ROX Premixed, Vazyme Q141). qRT-PCR reactions were performed on a StepOne plus Real-Time PCR System. Primers are listed in Table 1.

### Imaging

Cultured cells were observed using a Leica DMi8 inverted fluorescent microscope. Bright-field images of zebrafish embryos were taken using an Olympus SZ61 stereomicroscope. Fluorescent images were taken using a Leica TCS SP5 confocal microscope.

### Plasmids

Plasmids used in this study (and their origins): pmCherry-N1-KAT2A, pEGFP-N1-PAX6, pEGFP-N1-RNF8, pEGFP-N1-Trim11, pEGFP-N1-PAX6-K75R, pEGFP-N1-PAX6-K75Q, pEGFP-N1-PAX6-K264R, pEGFP-N1-PAX6-K264Q, pEGFP-N1-PAX6-K246Q, pEGFP-N1-PAX6-K75Q+264Q, pEGFP-N1-PAX6-K246Q+264Q, pET-22b(+)-PAX6, pET-22b(+)-KAT2A His-FLAG-RNF8, and His-FLAG-TRIM11. All plasmids were purchased from Anhui General Biology.

### Mass spectrometry analysis

For acetylation site analysis, pcDNA3.1(+)-stag-PAX6 was transfected into HEK293T cells and S-Tag-PAX6 was harvested by immunoprecipitation in 48 h using Anti-S-tag antibody (BIOSS, bs-33017R) and sent to Shanghai Applied Protein Technology Co, Ltd for mass spectrometry analysis on acetylated lysine.

Gel pieces were destained in 50 mM NH4HCO3 in 50% acetonitrile (v/v). Gel pieces were dehydrated with 100 μl of 100% acetonitrile for 5 min and rehydrated in 10 mM dithiothreitol and incubated at 56 °C for 60 min. Gel pieces were once again dehydrated in 100% acetonitrile and rehydrated with 55 mM iodoacetamide. Samples were incubated at room temperature in the dark for 45 min. Samples were washed with 50 mM NH4HCO3 and dehydrated with 100% acetonitrile. Samples were rehydrated with 10 ng/μl trypsin and resuspended in 50 mM NH4HCO3 on ice for 1 h. Samples were digested with trypsin at 37 °C for 20 h. Peptides were extracted with 50% acetonitrile/5% formic acid and then 100% acetonitrile. Peptides were lyophilized to completion and resuspended in 2% acetonitrile/0.1% formic acid. The tryptic peptides were dissolved in 0.1% formic acid (solvent A) and directly loaded onto a home-made reversed-phase analytical column (15 cm length, 75 μm i.d.). The gradient comprised an increase from 6% to 23% solvent B (0.1% formic acid in 98% acetonitrile) over 16 min, 23% to 35% in 8 min, and up to 80% in 3 min, then was kept at 80% for the last 3 min, all at a constant flow rate of 400 nl/min on an EASY-nLC 1000 UPLC system. The peptides were subjected to NSI source followed by tandem mass spectrometry (MS/MS) in Q ExactiveTM Plus (Thermo Fisher Scientific) coupled online to the UPLC. The electrospray voltage applied was 2.0 kV. The *m/z* scan range was 350 to 1800 for full scans, and intact peptides were detected in the Orbitrap at a resolution of 70,000. Peptides were then selected for MS/MS using NCE setting as 28, and the fragments were detected in the Orbitrap at a resolution of 17,500. A data-dependent procedure alternated between 1 MS scan and 20 MS/MS scans with 15.0 s dynamic exclusion. Automatic gain control was set at 5E4.

Based on the raw data, Mascot 2.2 was used to search the rat protein database uniprot_Rattus_norvegicus_36135_20200217/zjk_3_20200721 established by Shanghai Applied Protein Technology Co, Ltd with these parameters: Enzyme, Trypsin; Fixed modifications, Carbamidomethyl (C); Variable modifications, Oxidation (M)/Acetyl(K)/GlyGly(K)/Phospho(STY); Missed Cleavage, 2, Peptide Mass Tolerance, 20 ppm; Fragment Mass Tolerance, 0.1 Da; Filter by score ≥20.

### Data analysis

The number of cells were counted using Fiji (https://imagej.net/software/fiji) per imaged area. Bright field and fluorescence images were processed using Fiji (file conversions, brightness and contrast), Adobe Photoshop (image rotation), and Adobe Illustrator (alignment). Representative images were taken from measurements of at least three repeats. Proliferating or apoptotic cells in each imaged eye were counted using Fiji on z-stack images after maximum-projection, and data are presented as a total number of proliferating or apoptotic cells. In the cultured NSCs, the proliferating and apoptotic cells were counted and quantified in five areas in each sample, and the ratios were calculated. Blinding was performed by hiding grouping information when conducting measurement. Axon lengths were determined by measuring the distance from the cell body to the growth cone of the longest process.

Relative mRNA expression levels were normalized to endogenous GAPDH and calculated using the ΔΔCt method. Differences were determined comparing with control samples, which were treated with control siRNA or control Morpholino Oligonucleotides.

For Western blotting, protein expression was normalized to the levels of endogenous GAPDH or β-Actin. Negative controls used for Western blotting were control Morpholino Oligonucleotides for gene specific Morpholino Oligonucleotides, mCherry mRNA for PAX6 mRNA injections, DMSO for MB-3 treatment. Relative ubiquitinated PAX6 level was normalized by dividing the amount of ubiquitinated protein by the amount of PAX6.

All statistical analysis was performed in GraphPad Prism 8. Unless stated otherwise, all measurements are shown as mean ± SEM. Student’s *t* test, one-way ANOVA, and two-way ANOVA were used for comparisons. Following one-way ANOVA, Tukey’s multiple comparisons or Dunnett’s multiple comparisons were performed for comparison between every two groups or between one control group and all other groups, respectively. Following two-way ANOVA, Fisher’s LSD was performed for multiple comparisons. One-tailed *p* values are provided in statistical diagrams.

## Data availability

All data generated in this study are included in this article and the supporting information.

## Supporting information

This article contains supporting information.

## Conflict of interest

The authors declare that they have no conflicts of interest with the contents of this article.
