# Supplementary material for: Histone acetyltransferase KAT2A modulates neural stem cell differentiation and proliferation by inducing degradation of the transcription factor PAX6
Source: J Biol Chem. 2023 Feb 13;299(3):103020. doi: 10.1016/j.jbc.2023.103020 (PMC10011063; doi:10.1016/j.jbc.2023.103020)
Supplement: Supporting Figure S1–S5 [file mmc1.docx]

# Supplementary data


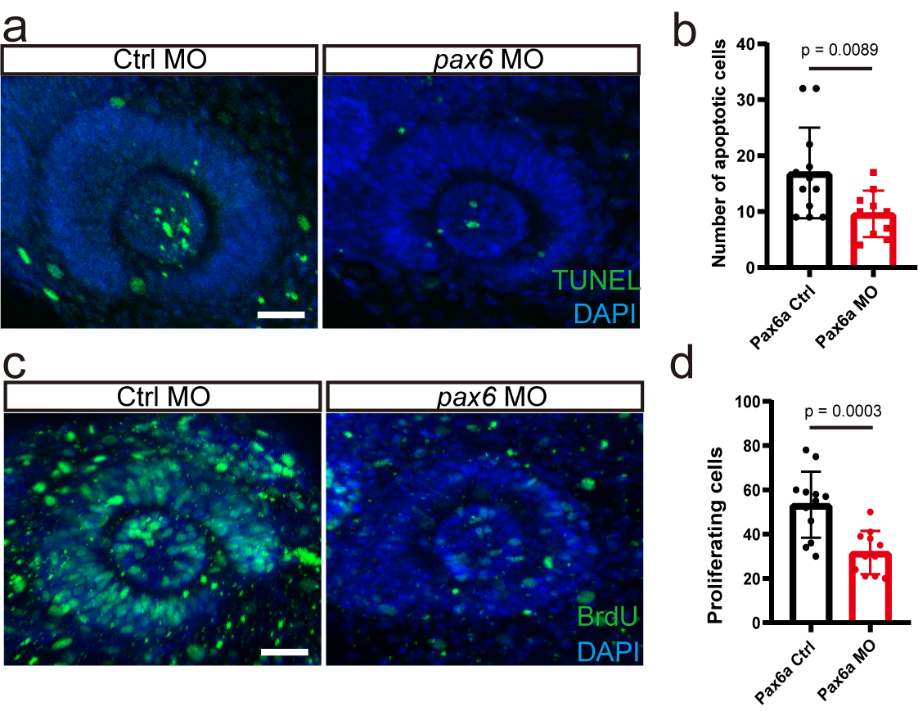


**Figure S1.** Apoptosis and proliferation were decreased in zebrafish embryos of *pax6* depletion. (a, b) TUNEL assay in *pax6* morphants showed a reduced number of apoptotic cells. Scale bar = 50 μm. n = 12 and 10 zebrafish, Student’s *t*-test. (c, d) BrdU assay in *pax6* morphants showed a reduced number of proliferating cells. Scale bar = 50 μm. n = 12 and 11 zebrafish, Student’s *t*-test.


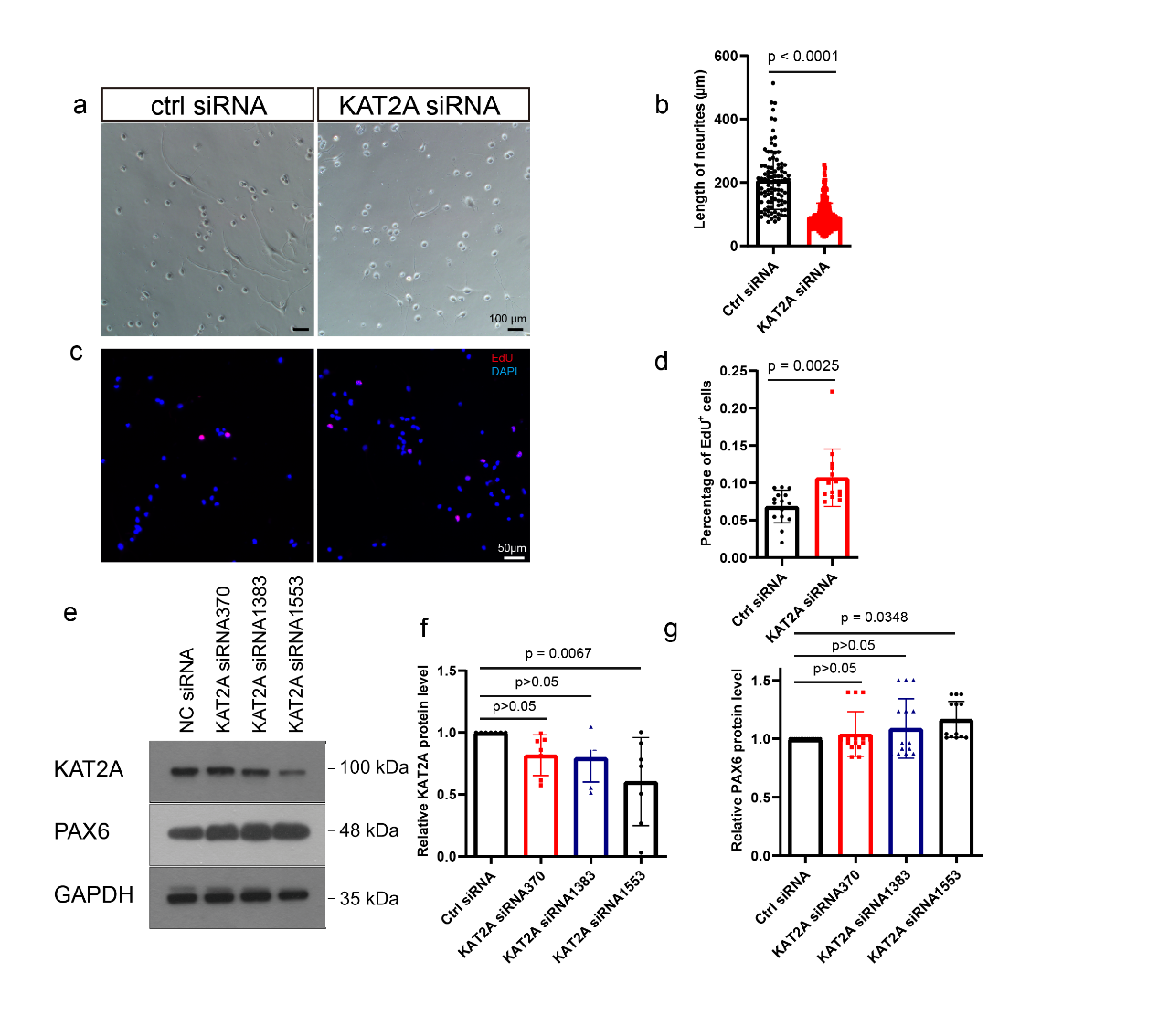


**Figure S2.** Differentiation and PAX6 expression in differentiating NSCs treated with KAT2A-specific siRNA. (a-d) Representative images and statistical diagrams of reduced process length and increased proliferating cells in differentiating NSCs treated with KAT2A siRNA for 24 h, 48 h, and 72 h, respectively. n = 96 and 359 cells for neurite length measurement, n = 15 and 14 dishes of cultured NSCs for EdU proliferation analysis, Student’s *t*-test. (e-g) Representative image and statistical diagrams of KAT2A and PAX6 protein levels detected by Western Blotting in differentiating NSCs treated with KAT2A-specific siRNA. For panel f, n = 7, 7, 7, 7 dishes of cultured NSCs, one-way ANOVA followed by Dunnett’s multiple comparison tests. For panel g, n = 15, 15, 15, 15 dishes of cultured NSCs, one-way ANOVA followed by Dunnett’s multiple comparison tests.


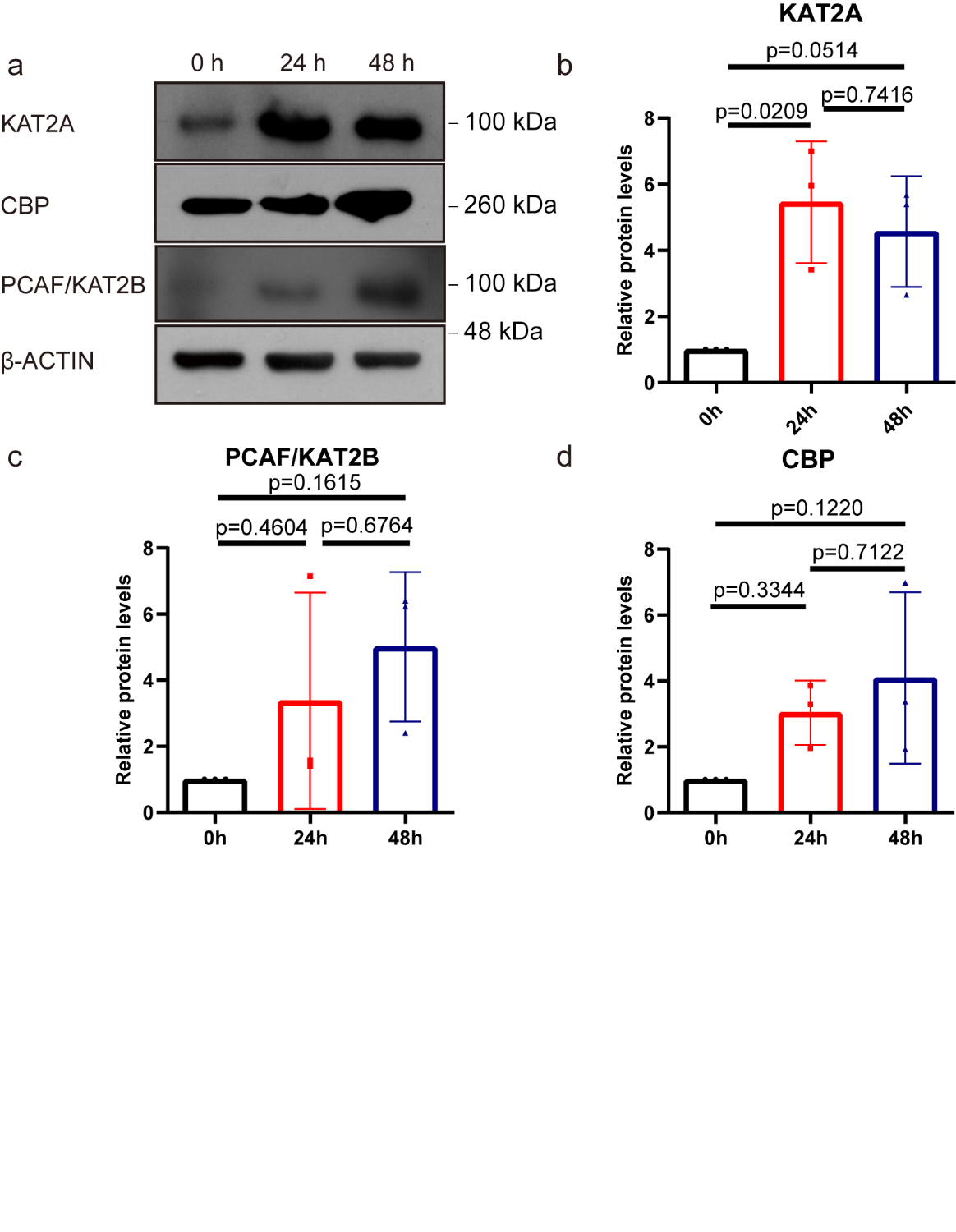


**Figure S3.** KAT2A, CBP, and PCAF are increased during the differentiation of NSCs. (a-d) Expressions of HATs including KAT2A, CBP, and PCAF in differentiating NSCs. n = 3 dishes of cultured NSCs, one-way ANOVA followed by Tukey’s multiple comparison tests.


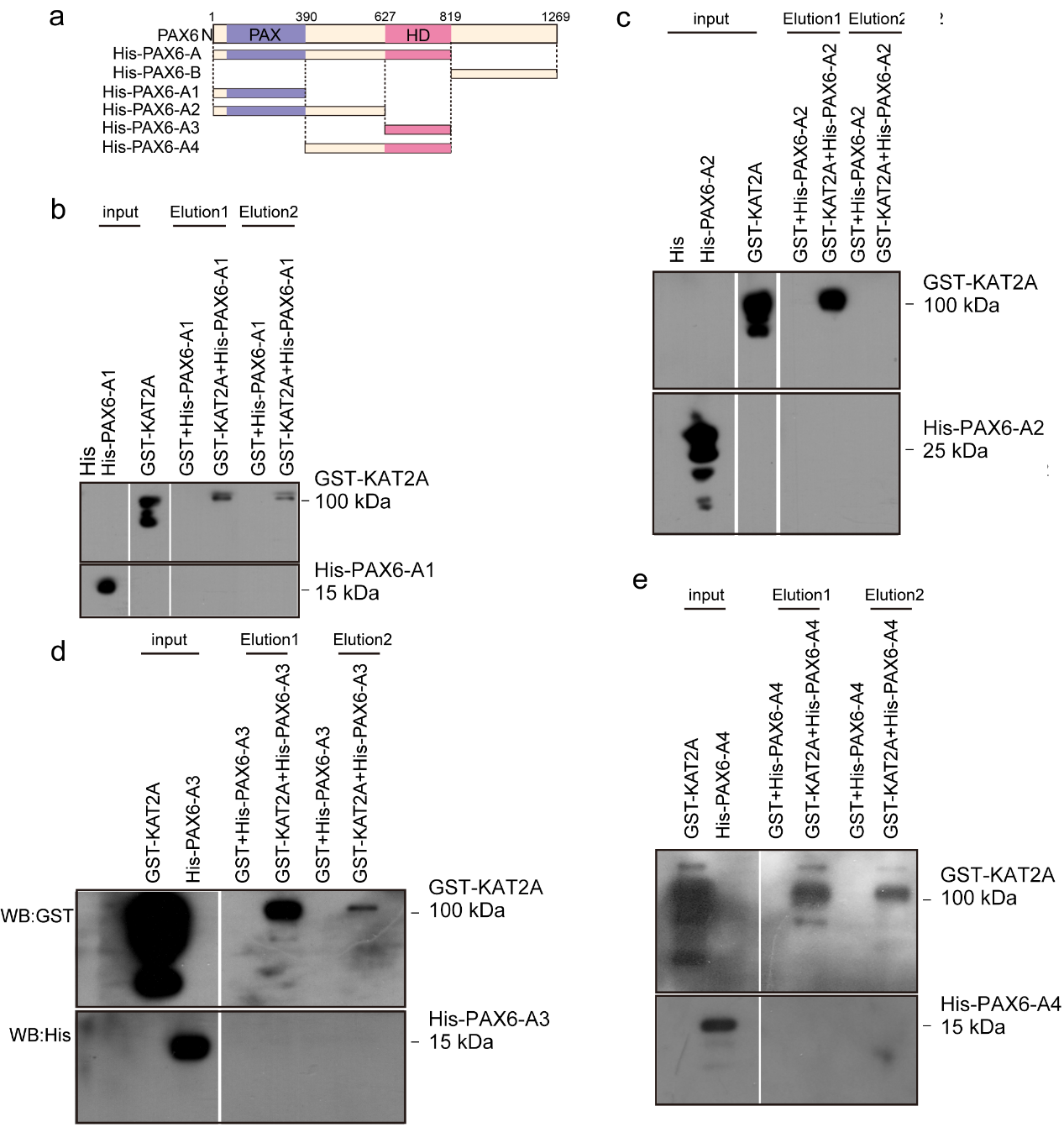


**Figure S4.** Analysis of the interaction between KAT2A and PAX6. (a) Diagram showing PAX6 truncated proteins used in this study. (b-e) Detection of the interaction between KAT2A and PAX6-A1/A1/A3/A4 truncated proteins using immunoprecipitation and Western blotting showed no interaction between KAT2A and these PAX6 truncated proteins.


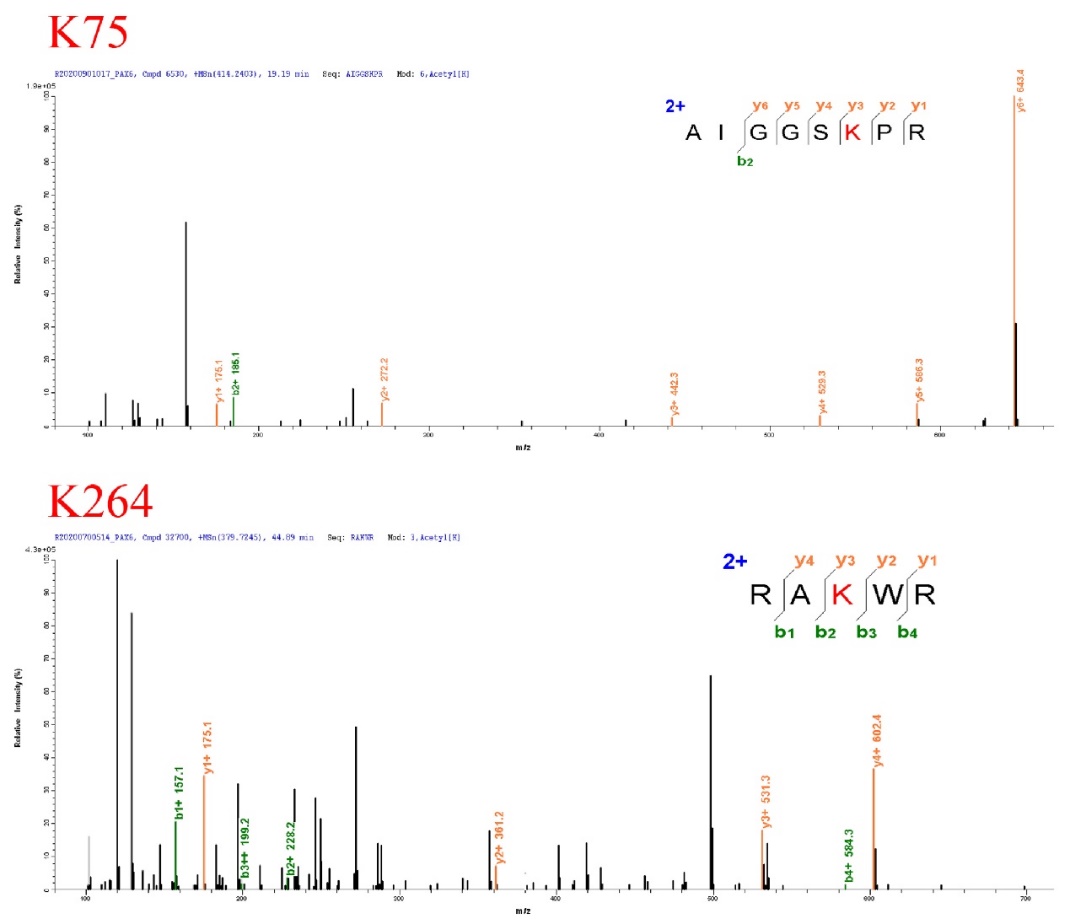


**Figure S5.** Mass spectrometry data of fragments containing predicted ubiquitination sites K75 and K264.
